# Interferon as an immunoadjuvant to enhance antibodies following influenza B infection and vaccination in ferrets

**DOI:** 10.1038/s41541-024-00973-2

**Published:** 2024-10-24

**Authors:** Thomas Rowe, Ashley Fletcher, Pavel Svoboda, Jan Pohl, Yasuko Hatta, Gabriela Jasso, David E. Wentworth, Ted M. Ross

**Affiliations:** 1 Influenza Division, Centers for Disease Control and Prevention, Atlanta, GA USA; 2 Department of Infectious Diseases, University of Georgia, Athens, GA USA; 3 Ampersand Biosciences, Lake Clear, NY USA; 4 Division of Core Laboratory Services and Response, Centers for Disease Control and Prevention, Atlanta, GA USA; 5 Center for Vaccines and Immunology, University of Georgia, Athens, GA USA; 6 Florida Research and Innovation Center, Cleveland Clinic, Port St. Lucie, FL USA; 7 Department of Infection Biology, Cleveland Clinic, Cleveland, OH USA

**Keywords:** Influenza virus, Influenza virus

## Abstract

Despite annual vaccination, influenza B viruses (IBV) continue to cause significant morbidity and mortality in humans. We have found that IBV infection resulted in a weaker innate and adaptive immune response than influenza A viruses (IAV) in ferrets. To understand and overcome the weak immune responses to IBV in ferrets, we administered type-I or type-III interferon (IFN) to ferrets following infection or vaccination and evaluated their effects on the immune response. IFN signaling following viral infection plays an important role in the initial innate immune response and affects subsequent adaptive immune responses. In the respiratory tract, IFN lambda (IFNL) has regulatory effects on adaptive immunity indirectly through thymic stromal lymphopoietin (TSLP), which then acts on immune cells to stimulate the adaptive response. Following IBV infection or vaccination, IFN treatment (IFN-Tx) upregulated gene expression of early inflammatory responses in the upper respiratory tract and robust IFN, TSLP, and inflammatory responses in peripheral blood cells. These responses were sustained following challenge or vaccination in IFN-Tx animals. Serum IFNL and TSLP levels were enhanced in IFN-Tx animals following challenge/rechallenge over mock-Tx; however, this difference was not observed following vaccination. Antibody responses in serum of IFN-Tx animals following IBV infection or vaccination increased more quickly and to higher titers and were sustained longer than mock-Tx animals over 3 months. Following rechallenge of infected animals 3 months post treatment, antibody levels remained higher than mock-Tx. However, IFN-Tx did not have an effect on antibody responses following challenge of vaccinated animals. A strong direct correlation was found between TSLP levels and antibody responses following challenge-rechallenge and vaccination-challenge indicating it as a useful tool for predicting adaptive immune responses following IBV infection or vaccination. The effects of IFN on strengthening both innate and adaptive responses to IBV may aid in development of more effective treatments following infection and improved influenza vaccines.

## Introduction

The vast majority of influenza studies focus on influenza A viruses (IAV), however, influenza B viruses (IBV) represent nearly one-quarter of all influenza infections and are generally responsible for most cases late in the influenza season^1^. Even though IBV co-circulate with IAV, much less is known about the innate and adaptive responses following IBV infection and vaccination^2,3^. IBV are not divided into subtypes as IAV, but instead are classified into two antigenically distinct lineages: B/Yamagata and B/Victoria^4^. Some differences between IBV demographics in humans indicate that patients with Victoria lineage are generally younger (0–4 years, 60% versus 40% in adults; 5–14 years, 72% versus 28% in adults) compared to patients infected with Yamagata lineage; however, clinical presentation is equal^5^. The average vaccine effectiveness for IBV is 54%^6^, however, during the 2014/2015 influenza season vaccine, it was 57%. During this period fewer than 20% of older children (9–17 years) had a 4-fold rise in antibody titer to the IBV component following vaccination^7^.

Ferrets are the primary animal model for influenza research and vaccine studies^8–11^. Therefore, ferrets were either vaccinated or infected with IBV to assess immune responses. In studies comparing multiple circulating IAV and IBV strains in ferrets, IBV induced weaker immune responses even though IBV strains replicated as well as IAV strains in the upper respiratory tract (URT)^12,13^. In addition, live attenuated influenza virus (LAIV) vaccines elicited weaker antibodies generated to the IBV components (B-Victoria and B-Yamagata) than to the IAV components (A/H1N1pdm09 and A/H3N2)^14^.

Following infection of respiratory epithelial cells, influenza viruses are recognized by the innate immune system using pattern recognition receptors (PRRs). These receptors recognize pathogen-associated molecular patterns (PAMPs) of which three classes of PRRs can recognize influenza virus infection and initiate an inflammatory response. The PRRs that recognize and activate innate signaling pathways to IAV include the toll-like receptors (TLRs), nod-like receptors and Retinoic acid Induced Gene 1 like receptor (RIG-I)^15^. TLR-3 is expressed in endosomes and recognizes viral double-stranded RNA which is produced during viral replication, while endosomal TLR-7 and cytoplasmic RIG-I recognize single-stranded RNA. These PAMP-PRR interactions lead to activation of several signaling pathways and to the production of type I and type-III interferons (IFNs) and inflammatory cytokines^16^. IFN signaling rapidly promotes an antiviral state by inducing IFN-stimulating genes to limit viral replication and spread and induces immune responses in neighboring cells to protect them from infection^17^. The non-structural protein 1 (NS1) of IAV is able to avoid the IFN response. NS1 has shown to be an IFN-antagonist to dampen host IFN response and facilitate virus replication^18^. In general, viruses avoid elements of the innate immune response, such as the IFN response, thereby allowing for increased virus replication and spread before the initiation of the adaptive immune response. The IAV NS1 inhibits type-I IFN release and can also inhibit dendritic cell (DC) maturation and function, which is important in adaptive immune responses^19^. Type-III IFN lambda (IFNL) can recruit DCs, regulate IL-10 production, and enhance type-I helper T cell responses^20^. IFNL is non-inflammatory and primarily activated in the mucosal tract and does not have the inflammatory effects seen with type-I IFNs. IFNL acts indirectly on DCs through the induction of thymic stromal lymphopoietin (TSLP) in the respiratory tract^21^, thus inducing a mucosal immune response. Upon induction TSLP regulates germinal center function and antigen-specific antibody responses^22^, thus having an important effect on the magnitude of the adaptive immune response.

Infection with IBV results in a delay in both innate and antibody responses in ferrets compared to IAV infection^13^. In this study, IFN was examined as an immunoadjuvant to enhance the immune responses in infected or vaccinated animals and assess protection following rechallenge with influenza. We used PEGylated ferret IFNs as potential immunoadjuvants following IBV infection or vaccination. Effects of IFNs as an alternative method to stimulate robust immune responses may be important to understanding reduced immune responses following IBV infection as well as a way to improve vaccine efficacy.

## Methods

### Interferon PEGylation and testing

Polyethylene glycol (PEG) attachment to molecules, PEGylation, is considered one of the most successful techniques to enhance the therapeutic and biotechnological potential of peptides and proteins^23–25^. Recombinant ferret type-I interferon alpha (IFNA) and type-III interferon lambda 3 (IFNL3) were purchased from Kingfisher Biotech, Inc. (Saint Paul, MN, USA). IFNA and IFNL3 were PEGylated with Hydroxyl-PEG-NHS ester 5000Da (HO-PEG5K-NHS; PEG5K; Sigma-Aldrich, Milwaukee, WI, USA) and m-PEG-Lys-NHS ester 20000Da (mPEG-Lys-NHS 20000; PEG20K; BroadPharm, San Diego, CA, USA). One microgram per microliter solutions of IFNA and IFNL3 were prepared in 100 mM pH 7.2 sodium phosphate buffer. Fifteen-fold molar excess of PEGs over the molar concentration of proteins were added to protein solutions. PEGs were dissolved in dimethyl sulfoxide (DMSO). The DMSO volume did not exceed 20% of total reaction volume. The mixtures were incubated at 25 °C for 1 h. One microgram aliquots of each sample were taken and electrophoresed on 4–12% NuPAGE Bis-Tris polyacrylamide gels and stained with Imperial Protein Stain to determine the extent of protein modification. Activity of PEGylated-IFNs was determined using an IFN bioassay. The modified proteins were stored at −80 °C prior to use in animal experiments.

### Viruses

Influenza B-Yamagata lineage virus (B/Phuket/3073/2013, “PH”) was used for infection and vaccination studies. For infection studies PH was passaged in Madin Darby Canine Kidney (MDCK) cells, to retain antigenic similarity to the original human isolate and to avoid structural changes in the hemagglutinin which could occur from passage in eggs^26–28^. The virus was passaged in MDCK cells “C#” according to established procedures^29^. PH passage C4 was used for all challenge and rechallenge experiments. For the vaccine study, live-attenuated influenza virus B-Yamagata lineage strain B/Phuket/3073/2013-CDC-LV11B (LAIV) was developed according to established procedures^30^ and passaged in embryonated chicken eggs^29^. All Viral titers, challenge/rechallenge and vaccine strain, were determined by focus-forming assay (FFA) and given as log focus forming units per milliliter (FFU/mL). Viral titers for challenge/rechallenge virus (PH) and vaccine strain (LAIV) used in this study were 10^7.54^ FFU/mL and 10^8.93^ FFU/mL respectively.

### Ferrets and study design

All animal procedures were approved by the Institutional Animal Care and Use Committee of the Centers for Disease Control and Prevention in an Association for Assessment and Accreditation of Laboratory Animal Care International accredited facility. Male fitch ferrets (Triple F Farms, Sayre, PA), between 1.2 and 2 years of age and seronegative for currently circulating influenza viruses were used for all experiments.

A pilot experiment (Experiment A) to determine the optimal time of addition of interferon followed by two separate experiments were conducted using 18 ferrets for each: (B) challenge-rechallenge experiment and (C) vaccination-challenge experiment (Table 1).

**Table 1:** Pilot study—timing of PEGylated-IFN administration

Group	Infection	Post-infection treatment	Treatment (hours post-infection)	*N*
1	B/Phuket/3073/2013	Mock	N/A	3
2		PEG-IFNα	8	2
			24	2
			48	2
3		PEG-INFλ3	8	2
			24	2
			48	2

Ferret study—experimental design. Pilot study to determine timing of PEG-IFN addition following IBV challenge. Mock-infected/un-treated (*N* = 3 animals), PEG-IFNA Treated at 8 h, 24 h, and 48 h post challenge (*N* = 2/group). PEG-IFNL treated at 8 h, 24 h, and 48 h post challenge (*N* = 2/group). NW samples collected prechallenge (D0) and D1, 3, 5, 7 post challenge for virus titration by FFA.

#### Pegylated-interferon (PEG-IFN) time of addition—pilot study

In this pilot study, we performed a small study of 2 ferrets/group to determine the optimal time of addition of PEGylated-IFN so as to not affect virus replication. All ferrets (*N* = 15) were challenged intranasally with 4 × 10^5^ FFU of B/Phuket/3073/2013 and treated intranasally with PEG-IFN or PEG-IFNL at 8 h, 24 h or 48 h post challenge (*N* = 2 ferrets/group). An untreated (Mock) group (*N* = 3 ferrets) was included as a mock control as a baseline group. Nasal wash (NW) samples were collected prechallenge and Days 1, 3, 5, and 7 post challenge and assessed for viral load by FFA.

Prior to challenge, the ferrets were first anesthetized with an intramuscular cocktail of ketamine (15–30 mG/kG) plus xylazine (1–2 mG/kG) “KX” and challenged intranasally with 1 mL of virus (4 × 10^5^ FFU/mL) or PBS. At various timepoints post challenge, ferrets were anesthetized and 1 μG/kG PEGylated-IFN (IFNA or IFNL) was introduced intranasally to the appropriate groups.

#### Challenge-rechallenge study

In this study, we chose a representative virus (B/Phuket/3073/2013, “PH”), which in previous experiments typified the delayed and low immune responses of IBVs, for a challenge/rechallenge study. Six groups of three ferrets per group were used: Mock-challenged/mock-treated (UN-MOCK), mock-challenged/IFNA-treated (UN-IFNA), mock-challenged/IFNL3-treated (UN-IFNL), PH-challenged/mock-treated (PH-MOCK), PH-challenged/IFNA-treated (PH-IFNA), and PH-challenged/IFNL3-treated (PH-IFNL). The ferrets were first anesthetized with an intramuscular cocktail of ketamine (15–30 mG/kG) plus xylazine (1–2 mG/kG) “KX” and challenged intranasally with 1 mL of virus (4 × 10^5^ FFU/mL) or PBS. Twenty-four hours post-challenge ferrets were anesthetized and 1 μG/kG PEGylated-IFN (IFNA or IFNL) or PBS was introduced intranasally to the appropriate groups. The animals were monitored over 28 days post challenge for weight loss, fever, and other clinical signs (lethargy, sneezing, and dyspnea) of infection. The animals were weighed on day 0 prior to PH challenge to establish a baseline weight and then daily for the first 10 days post challenge followed by weekly thereafter through day 28. Changes in weight were calculated as percentage loss or gain from day 0 weight. For fever calculation, a temperature transponder [IPTT-300; Bio Medic Data Systems (BMDS), Waterford, WI, USA] was programmed to identify each ferret and implanted subcutaneously between the shoulder blades of each animal and read with a scanner (DAS-6007 IPTT Scanner System, BMDS). A baseline temperature (°F) was determined by the average temperature over 3 days prior to challenge. Temperatures were assessed over the first 10 days post challenge, then weekly and fever was determined as any temperature greater or equal to 2 °F above baseline. Ferrets were assessed daily for clinical signs within a 2-h window between 9 AM and 11 AM during peak normal activity and to reduce variation due to circadian rhythms^31^. Lethargy was determined by the relative inactivity index (RII) from day 0 through day 7 post challenge; this period allowed for lethargy calculation throughout the period of active replication in the ferret respiratory tract. RII, sneezing and dyspnea were assessed each day prior to handling and sedation of the animals. Clinical signs assessment was consistent with previously established methods^32,33^. Ferrets were monitored for 12 weeks post challenge and either challenged (UN-MOCK, UN-IFNA, and UN-IFNL groups) or rechallenged (PH-MOCK, PH-IFNA, and PH-IFNL groups) intranasally with 1 mL PH (4 × 10^5^ FFU/mL). Ferrets were monitored for 7 days post challenge/rechallenge as listed above. Study design illustrated in Table 1B.

#### Vaccination-challenge study

The live-attenuated B-Yamagata lineage strain B/Phuket/3073/2013-CDC-LV11B (LAIV) was used as the vaccine for this study. Six groups of three ferrets per group were used for the vaccination-challenge study: Mock-vaccinated/mock-treated (UN-MOCK), mock-vaccinated/IFNA-treated (UN-IFNA), mock-vaccinated/IFNL3-treated (UN-IFNL), vaccinated/mock-treated (LAIV-MOCK), vaccinated/IFNA-treated (LAIV-IFNA), and vaccinated/IFNL3-treated (LAIV-IFNL). The ferrets were first anesthetized with KX and vaccinated intranasally with 1 mL LAIV (4 × 10^5^ FFU/mL) or PBS. Twenty-four hours post-vaccination ferrets were anesthetized and 1 μG/kG PEGylated-IFN (IFNA or IFNL) or PBS was introduced intranasally to the appropriate groups. Animals were monitored as in challenge-rechallenge study. At 12 weeks post vaccination, all animals were challenged intranasally with 1 mL PH (4 × 10^5^ FFU/mL). Ferrets were monitored for 7 days post challenge as described previously. Study design illustrated in Table 1C.

### Ferret sample collection and processing

Pre-challenge and on days (D) 1, 3, 5, and 7 post challenge or vaccination, NW was collected from ferrets for virologic and gene expression. Additional NW samples (D0, D1, D3, D5, and D7) were collected after 3 months for the challenge-rechallenge study or the vaccination-challenge study. These collection timepoints correspond to typical viral kinetics observed in the URT of ferrets infected with seasonal influenza viruses^34,35^. Animals were sedated with KX followed by flushing the nares by instillation of 2 mL NW solution (PBS, 1% BSA, antibiotics), inside a class-II biosafety cabinet, and collected when expelled in a Petri dish. NW was centrifuged (5 min, 2500 × *g* at 4 °C), supernatant was stored at −80 °C for virus titration. One hundred microliters (μL) PBS was added to NW pellets followed by 280 μL of AVL lysis buffer. Samples were mixed 3–4 times and lysate was frozen at −80 °C until RNA extraction. Carrier RNA was added to each sample and RNA was extracted using EZ1 DSP kit on a Qiagen EZ1 Advanced XL extractor according to the manufacturer’s instructions (Qiagen, Germantown, MD, USA). RNA was eluted in 120 μL RNase-free water and stored at −80 °C until evaluated for gene expression by quantitative real-time PCR (qRT-PCR).

Blood samples were collected for gene expression (pre-challenge, D3 and D5; re-challenge D0, D1, D3, and D5) and antibody/cytokine analysis (Pre-challenge, weeks 1, 2, 4, 8, 12, and D7 post-rechallenge). Blood was collected from the cranial vena cava and transferred to an SST tube (serum for antibody and cytokine analysis) and a K2/EDTA tube (cells for gene expression). Serum was separated from SST tubes by centrifugation for 10 min at 1500 × *g* at room temperature. For purification of peripheral blood mononuclear cells (PBMC), an equal volume of PBS was added to blood in K2/EDTA tubes and overlayed onto Histopaque®-1077 (Sigma, St. Louis, MO, USA). Samples were centrifuged with no brake at 900 × *g* for 20 min at room temperature. Cells were collected from the Histopaque® interface, washed with cell culture media (RPMI-1640, 10% heat-inactivated fetal bovine serum (FBS)) and treated with ammonium chloride to lyse any remaining red blood cells. The final cell pellet was resuspended in 1 mL cell culture medium. Two hundred eighty microliters of AVL lysis buffer was added to 200 μL PBMC samples in cell culture medium obtained by density gradient centrifugation over Ficoll-Hypaque. Samples were mixed 3–4 times and lysate was frozen at −80 °C until RNA extraction. Carrier RNA was added to each sample and RNA was extracted using EZ1 DSP kit on a Qiagen EZ1 Advanced XL extractor according to the manufacturer’s instructions (Qiagen, Germantown, MD, USA). RNA was eluted in 120 μL RNase-free water and stored at −80 °C until evaluated for gene expression by qRT-PCR. The remaining PBMC sample was cryopreserved in FBS, 10% DMSO and stored in the vapor phase of liquid nitrogen.

### Virus kinetics assessment by focus forming assay

NW samples collected from influenza virus-infected and vaccinated ferrets were tested for virus with an FFA, as previously described^36^. Briefly, ½-log serially diluted influenza viruses in virus growth medium plus trypsin (DMEM, 0.1% BSA, 1 μG/mL TPCK-treated trypsin (Sigma, St. Louis, MO, USA)) were added to confluent monolayers of MDCK-SIAT1 cells^37^ in 96-well flat-bottom tissue culture plates in quadruplicate. Following a 2-h incubation at 37 °C, and overlay of 1.2% Avicel RC/CL^38^ (Type: RC581 NF; FMC Health and Nutrition, Philadelphia, PA, USA) in 2X MEM containing 1 μG/mL TPCK-treated trypsin, 0.1% BSA, and antibiotics] was added. Plates were incubated overnight at 37 °C, 5% CO_2_, fixed, permeabilized, and stained with a monoclonal antibody pool to influenza B nucleoprotein (International Reagent Resource; www.internationalreagentresource.org). Infectious foci (spots) were visualized using TrueBlue substrate (Sera Care, Inc., Milford, MA, USA). The foci enumerated using a CTL Bio Spot Analyser with ImmunoCapture 6.4.87 software (CTL, Shaker Heights, OH, USA). The FFA titer was determined by multiplying sample dilution which gave between one hundred and three hundred spots by the spot number at that dilution, to obtain the Focus Forming Units per milliliter (FFU/mL). The foci in the cell control (CC) were subtracted and number of foci remaining was multiplied by twenty to give FFU/mL. The limit of detection was 10^1.3^ FFU/mL.

### Neutralizing antibody responses by focus reduction assay

The focus reduction assay (FRA), initially developed by the WHO Collaborating Centre in London, United Kingdom, was modified and utilized in this study as previously described^13^. Two-fold serially diluted Receptor Destroying Enzyme—treated sera^29^ from vaccinated or infected ferrets was added to 96-well plates containing confluent MDCK-SIAT1 cells^37^. Afterwards, standardized B/Phuket/3073/2013 virus was added to each plate or media to CC wells. The virus was standardized by FFA^36^ to determine the FFU/mL. Following a 2 h incubation, an overlay containing Avicel^38^ and 1 μG/mL TPCK-treated trypsin, 0.1% BSA, and antibiotics was added to each well. Plates were incubated for overnight at 37 **°**C, 5% CO_2_. The overlays were removed, washed with PBS, fixed with ice-cold 4% (w/v) paraformaldehyde in PBS (10% formalin), and permeabilized with 0.5% Triton X-100 in PBS/glycine. An incubation with primary antibody pool against influenza B nucleoprotein^39^ (www.internationalreagentresource.org) was performed, washed, followed by an incubation with secondary goat anti-mouse peroxidase-labeled IgG (Sera Care, Inc., Milford, MA, USA). Plates were washed and infectious foci (spots) were visualized using TrueBlue substrate (Sera Care, Inc., Milford, MA, USA) containing 0.03% H_**2**_O_**2**_. Plates were rinsed with deionized water, dried, and foci enumerated using a CTL Bio Spot Analyser with ImmunoCapture 6.4.87 software (Cellular Technology Ltd., Shaker Heights, OH, USA). The FRA titer was reported as the reciprocal of the highest dilution of serum corresponding to 50% foci reduction compared to the virus control (VC) minus the CC.

### Gene expression in ferret cells

Ferret primers generated to pro-inflammatory (*MCP1*, *IL-1B*, and *IL-6)*, TH1 (*CXCL10*), TH2 directed (*IL-2*), T-regulatory (*TGFB1*), T-effector (*IL-4*, *IL-12p40*, and *IL-17*), apoptosis (*Granzyme A*), Type-I/II/III interferons (*IFNA*, *IFNB*, *IFNG*, and *IFNL3*), interferon responses (*STAT1*, *STAT2*, *STAT3*, *RIG-I*, *SOCS3*, and *TSLP)* and housekeeping (*GAPDH*) genes were used. For genes using TaqMan, probes were modified with 6-FAM, fluorescein amidites (FAM) fluorophore on the 5′ end and a non-fluorescent Black Hole Quencher®-1 (BHQ-1) on the 3′ end. Additionally, a locked nucleic acid at adenine <LNA A> was incorporated in all probes in order to increase template binding strength for real-time PCR^40^. Primers and probes for all ferret genes were generated from published ferret sequences^36,41–43^. All primers and probes used in these experiments were generated in a previous study^36^; listed in Supplementary Table S1.

qRT-PCR was performed using an ABI 7500 Fast Dx Real-Time PCR instrument (Applied Biosystems, Waltham, MA, USA). PCR reactions were performed in a 5 μL RNA reaction volume using SYBR™GreenER™ qPCR SuperMix (Applied Biosystems) or SuperScript™ III Platinum™ One-Step qRT-PCR Kit for TaqMan reactions (InvivoGen, San Diego, CA, USA). An RT reaction for 30 min at 50 °C, inactivation for 2 min at 95 °C, followed by 40 amplification cycles at an annealing temperature of 50 °C. Reactions were performed on three ferrets for each virus and timepoint and the values were normalized by subtracting the mean value of the cycle threshold (C_T_) from that of the C_T_ for glyceraldehyde-3-phosphate dehydrogenase (GAPDH) housekeeping gene (ΔC_T_). The relative levels of gene expression for infected cells were determined by subtracting the individual ΔC_T_ values from that of average ΔC_T_ values of pre-infection cells (ΔΔC_T_) and expressing the final quantification values (2^−ΔΔCT^) as relative fold changes. Genes upregulated >10^4^-fold (>10,000) were given a value of 10,000 and genes downregulated <10^−4^-fold (<0.0001) were given a value of 0.0001.

### Assessment of cytokines and chemokines in ferret sera

Sera were collected from ferrets on day 0 (pre-challenge/vaccination) and weekly (1, 2, 4, 8, and 12) post challenge/vaccination as well as one week following 3-month re-challenge, challenge, or vaccination (R7). Samples were stored at −80 °C until testing by multiplex, bioassay, or ELISA as described below. All cytokine/chemokine values were normalized to pre-challenge/vaccination (day 0) to account for basal levels of each analyte and to observe effects of IBV and adjuvants.

Type-I/II IFN (IFNB and IFNG), TH1/TH2 (IP-10 and IL-2), pro-inflammatory (MCP-1, MIP-1B, TNFA, IL-6, IL-8, and IL-23) and T-effector (IL-4, IL-17, IL-12p40, and IL-12p70) levels in ferret sera were tested using a multiplex Luminex Assay kit [Ampersand Biosciences (www.ampersandbio.com) Lake Clear, NY, USA] containing ferret-specific antibodies and proteins in a microsphere-based assay and consisting of antigen-specific antibodies covalently coupled to magnetic Luminex beads and biotinylated detection antibodies in a capture-sandwich format as previously described^36^.

The assay was analyzed using a Luminex 200 Analyzer [Luminex Corporation (www.luminexcorp.com), Austin, TX, USA]. The cytokine/chemokine results were determined by extrapolating the analyte concentration from the measured mean fluorescence intensity value using the standard curve. Results were generated and extracted using the RBM Plate Reader and Plate Viewer analysis software, respectively. The limit of detection was 10 pg/mL. Data were exported with values represented to two significant figures and analyzed in GraphPad Prism 10 (GraphPad Software, La Jolla, CA, USA).

Ferret Type-III IFN (IFNL) was detected in serum using a HEK-Lambda reporter cell assay (HEK-Blue™ cells, InvivoGen, San Diego, CA, USA) and calculated from a standard curve using recombinant ferret IFNL3 [Kingfisher Biotech (www.kingfisherbiotech.com), St. Paul, MN, USA]. This assay was modified and tested for detection of ferret IFNL^36^. The limit of detection was 100 pg/mL.

TSLP was detected in ferret sera using a commercially available human TSLP Quantikine® ELISA Kit [R&D Systems (www.rndsytems.com), Minneapolis, MN, USA] according to manufacturer’s instructions. The limit of detection was 10 pg/mL. Ferret TSLP protein sequence available (NCBI BLAST Accession number XP_044943037.1) showed a 62.7% sequence homology to human TSLP protein (NCBI BLAST Accession number AAH40592.1); however, the homology may be greater since the ferret sequence was of low quality.

### Statistical analysis

GraphPad Prism 10 was used for all statistical analyses (GraphPad Software, La Jolla, CA, USA). One-way ANOVA was used to determine differences over time between groups and significance between groups was determined by 2-way ANOVA analysis. Gene expression analysis was performed from three ferrets per group. Spearman (1-tailed, 95% confidence) correlation method used for comparison of protein concentration (multiplex, bioassay, or ELISA) and antibody levels (FRA) for each treatment group to observe an increase or decrease in association. Multiple student’s *t* tests were used to assess the statistical differences between timepoints and treatment groups. A *p* value of <0.05 was considered statistically significant: *p < 0.05, **p < 0.01, ***p < 0.001, ****p < 0.0001.

## Results

### Virus replication kinetics is affected by timing of PEGylated-IFN administration following IBV infection in ferrets

Recombinant ferret IFNA and IFNL3 were PEGylated and used as immunoadjuvants in challenge-rechallenge and vaccination-challenge studies. PEGylation of the proteins was successful as demonstrated by bonding patterns of PEG to ferret IFNs, as visualized by imperial and silver staining, and retained activity following storage at 4 °C or freeze/thaw following storage at −80 °C (Supplementary Fig. 1). Even though PEG-IFNL3 activity was determined (Supplementary Fig. 1D), PEG-IFNA activity could not be determined using HEK-Blue™ Type I interferon reporter cells in a bioassay due to inability to detect Type I ferret IFN, as well as cross-reactivity of type-III IFNs in the assay (Supplementary Fig. 1C).

**Fig. 1:**
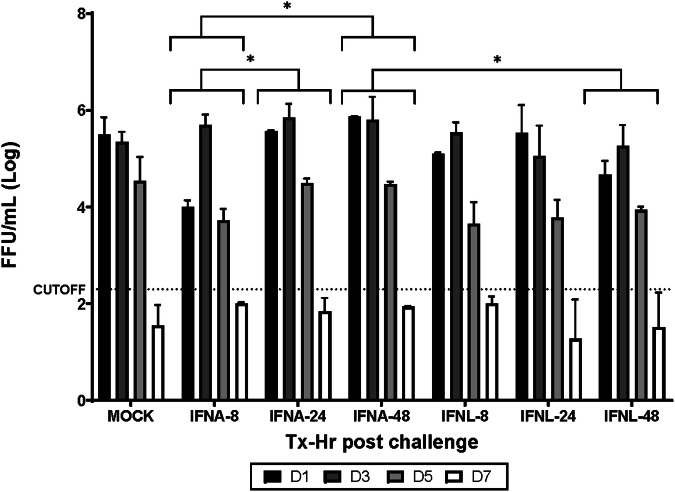
PEGylated IFN optimal time of addition following IBV challenge. Kinetics of virus replication following IBV challenge and addition of PEGylated IFN. PEGylated IFNA added at 8 h (IFNA-8), 24 h (IFNA-24), or 48 h (IFNA-48) post B/Phuket/3073/2013 challenge and compared to Mock treated group. PEGylated IFNL3 added at 8 h (IFNL-8), 24 h (IFNL-24) or 48 h (IFNL-48) post challenge. NW titers determined on D1, D3, D5, and D7 post challenge (bar color indicating time of NW collection) and tested for virus Log FFU/mL by FFA. Significance between groups tested by 2-way ANOVA. Significant differences between IFNA-8 and IFNA-24 (*p* = 0.0470) and IFNA-8 to IFNA-48 (*p* = 0.0154) as well as IFNA-48 to IFNL-48 (*p* = 0.0130). No significant differences noted between any groups with IFN added at 24 h and no significance compared to Mock-treated group.

A preliminary study was performed to determine the optimal time to add PEG-IFN post IBV challenge, 12 adult ferrets were intranasally challenged with PH followed by intranasal treatment with PEGylated-IFNA or -IFNL at 8 h, 24 h, or 48 h post-infection. Administration of IFNA at 8 h post infection resulted in a significant reduction in influenza virus kinetics compared to addition at 24 h (*p* = 0.047) or 48 h (*p* = 0.0154) post-infection (Fig. 1). Significant differences in virus titers were also observed at 48 h after IFNA and IFNL addition following IBV infection (*p* = 0.013). Overall, administration of IFNs at 24 h post-infection was the optimal time to treat ferrets and minimize the effects on virus replication (Tables 2 and 3).

**Table 2:** Challenge-rechallenge study

Group	Infection with influenza B virus	Treatment	*N*	Challenge with influenza B virus
	Day 0	Day 1 (24 h)		Day 84
UN-MOCK	Mock	Mock	3	B/Phuket/3073/2013
UN-IFNA		PEG-IFNA	3	
UN-IFNL		PEG-IFNL	3	
PH-MOCK	B/Phuket/3073/2013	Mock	3	
PH-IFNA		PEG-IFNA	3	
PH-IFNL		PEG-IFNL	3	

Challenge/rechallenge study (*N* = 3 ferrets/group). Animals treated with IFNA, IFNL, or Mock-treated 24 h post IBV challenge or post Mock challenged. NW collected pre challenge and D1, 3, 5, 7 post challenge and post challenge (D0, D1, D3, D5, and D7). Blood collected pre challenge, and D3, D5, Wk1, Wk2, Wk4, Wk8, and Wk12 post challenge and D7 post rechallenge. Animals rechallenged 3Mo post challenge.

**Table 3:** Vaccination-challenge study

Group	Vaccination with LAIV	Treatment	*N*	Challenge with wildtype virus
	Day 0	Day 1 (24 h)		Day 84
UN-MOCK	Mock	Mock	3	B/Phuket/3073/2013
UN-IFNA		PEG-IFNA	3	
UN-IFNL		PEG-IFNL	3	
Live-MOCK	B/Phuket/3073/2013-CDC-LV11B	Mock	3	
Live-IFNA		PEG-IFNA	3	
Live-IFNL		PEG-IFNL	3	

Vaccination/challenge study (*N* = 3 ferrets/group). Animals treated with IFNA, IFNL, or Mock-treated 24 h post Vaccination with LAIV vaccination or unvaccinated. NW collected pre vaccination and D1, 3, 5, 7 post vaccination and post challenge (D0, D1, D3, D5, and D7). Blood collected pre vaccination, and D3, D5, Wk1, Wk2, Wk4, Wk8, and Wk12 post vaccination and D7 post challenge. Animals challenged 3Mo post vaccination.

### IFN treatment reduced morbidity in ferrets following IBV challenge and re-challenge

The addition of PEGylated-IFN as an immunoadjuvant following influenza virus infection stimulates both innate and adaptive immune responses. IFN-treated ferrets showed reduced clinical signs (RII and or detectable virus in the URT over 7 days of observation (Table 4A). Three months post challenge, animals were either rechallenged (PH groups) or challenged (UN groups) with PH and assessed for 7 days (Table 4B). The mock-challenged animals (UN-MOCK/IFNA/IFNL) did not have any detectable lethargy, fever, or weight loss, regardless of treatment. Following virus challenge, IFN (PH-MOCK) had signs of severe morbidity. The untreated ferrets were lethargic (RII = 1.18) compared to IFNA-treated (RII = 1.14) and IFNL-treated (RII = 1.01) ferrets. Additionally, PH-MOCK ferrets were observed sneezing on D2 and D3 post challenge. However, although PH-IFNL-treated ferrets had no observable clinical signs (RII = 1.01), both the IFNL- and mock-treated ferrets developed fever at D2 post-challenge. Additionally, the IFNL-treated ferrets had the greatest weight loss (3.2%) post-challenge, even though the animal activity was normal. Interestingly, no fever was observed in the IFNA-treated animals. Following challenge, IFNA-treated ferrets had significantly higher viral NW titers in the URT than un-treated ferrets (*p* = 0.0223). In contrast, there was no significant difference in viral titers between PEG-IFNA and PEG-IFNL-treated animals (Table 4A). Following rechallenge there were no significant differences in viral titers between ferrets any group (Table 4B). Ferrets, previously treated with IFNs, and subsequently challenged had no differences in observed clinical signs, except in MOCK-treated ferrets that had fever (Table 4B; UN-Tx groups). Both untreated (UN-MOCK) and IFNA treated (UN-IFNA) developed fever on D2, however, no fever was observed following IFNL treatment (UN-IFNL) (Supplementary Fig. 2A–D). Taken together, IFN-treated ferrets had overall fewer signs of lethargy and had more rapid recovery from infection than untreated animals.

**Table 4:** Clinical signs and virus replication in IFN-treated ferrets following IBV challenge-rechallenge

	Group-Tx		Inactivity^a^ RII	Days sneezing	Average weight loss^b^	Average fever^c^	NW viral load^d^ (Avg ± SD Log_10_ FFU/mL)			
			(D1–7)	(%)	(D1–7)	(Max day)	D1	D3	D5	D7
A) Challenge		UN-MOCK	1.00	-	-	+2.1 °F (D1)	N/A	N/A	N/A	N/A
		UN-IFNA	1.00	-	-	-	N/A	N/A	N/A	N/A
		UN-IFNL	1.00	-	-	-	N/A	N/A	N/A	N/A
		PH-MOCK	1.18	D2–D3 (33)	0.6%	+2.3 °F (D2)	3.51 ± 0.95	5.37 ± 0.23	4.27 ± 0.67	1.30 ± 0.00
		PH-IFNA	1.14	-	1.2%	-	4.97 ± 0.47	5.45 ± 0.43	4.34 ± 0.53	2.26 ± 0.83
		PH-IFNL	1.01	-	3.2%	+2.9 °F (D2)	4.82 ± 0.31	5.38 ± 0.02	4.03 ± 0.06	1.80 ± 0.86
B) Challenge/rechallenge (3MO)		UN-MOCK	1.02	D2 (33)	3.3%	+3.2 °F (D2)	5.50 ± 0.36	5.34 ± 0.21	4.54 ± 0.49	1.55 ± 0.43
		UN-IFNA	1.04	D2 (33)	6.6%	+2.5 °F (D2)	5.32 ± 0.42	5.42 ± 0.38	4.83 ± 0.28	1.74 ± 0.16
		UN-IFNL	1.04	D2 (66)	1.6%	-	5.49 ± 0.33	5.53 ± 0.50	4.97 ± 0.38	2.13 ± 0.25
		PH-MOCK	1.05	D2–D3 (33)	0.1%	-	2.35 ± 0.21	2.03 ± 0.38	1.82 ± 0.46	2.24 ± 0.26
		PH-IFNA	1.01	-	-	-	2.12 ± 0.40	2.57 ± 0.33	2.35 ± 0.34	2.42 ± 0.44
		PH-IFNL	1.00	-	-	-	2.08 ± 0.62	2.30 ± 0.88	1.92 ± 0.48	2.47 ± 0.51

Clinical signs and virus replication in the URT of ferrets challenged with B/Phuket/3073/2013 (PH) followed by IFN treatment (or Mock) on D1 post challenge. Group designated as “UN” were mock challenged and groups designated as “PH” were challenged with IBV (PH). A) Initial challenge clinical signs and virus replication. Groups above the dotted line were mock-challenged with PBS and groups below the dotted line were challenged with PH. NW viral loads listed as N/A for mock-challenged groups. B) At 3 months post challenge, all groups were challenged (above the dotted line, UN) or rechallenged (below the dotted line, PH) with PH.

^a^Relative inactivity index “RII” (D1–D7) post challenge. RII=${\sum }_{(D1-7)}\left[{score}+1\right]n/{\sum }_{(D1-7)}n$.

^b^Day post challenge of peak weight loss percentage (day) from pre-challenge weight from D1–D7 post challenge.

^c^Average elevated temperature of at least +2 °F (Fever) above baseline (days post challenge elevated). Fever indicated by any temperature greater than the average baseline (D-2 to D0 pre-challenge) +2 °F.

^d^Average viral load in ferret URT on D1 through D7 post challenge. Baseline = 1.30 Log_10_ FFU/mL.

### IFN treatment induced an early inflammatory response in ferret URT and a robust, sustained IFN and inflammatory responses in PBMC following IBV challenge

**Fig. 2:**
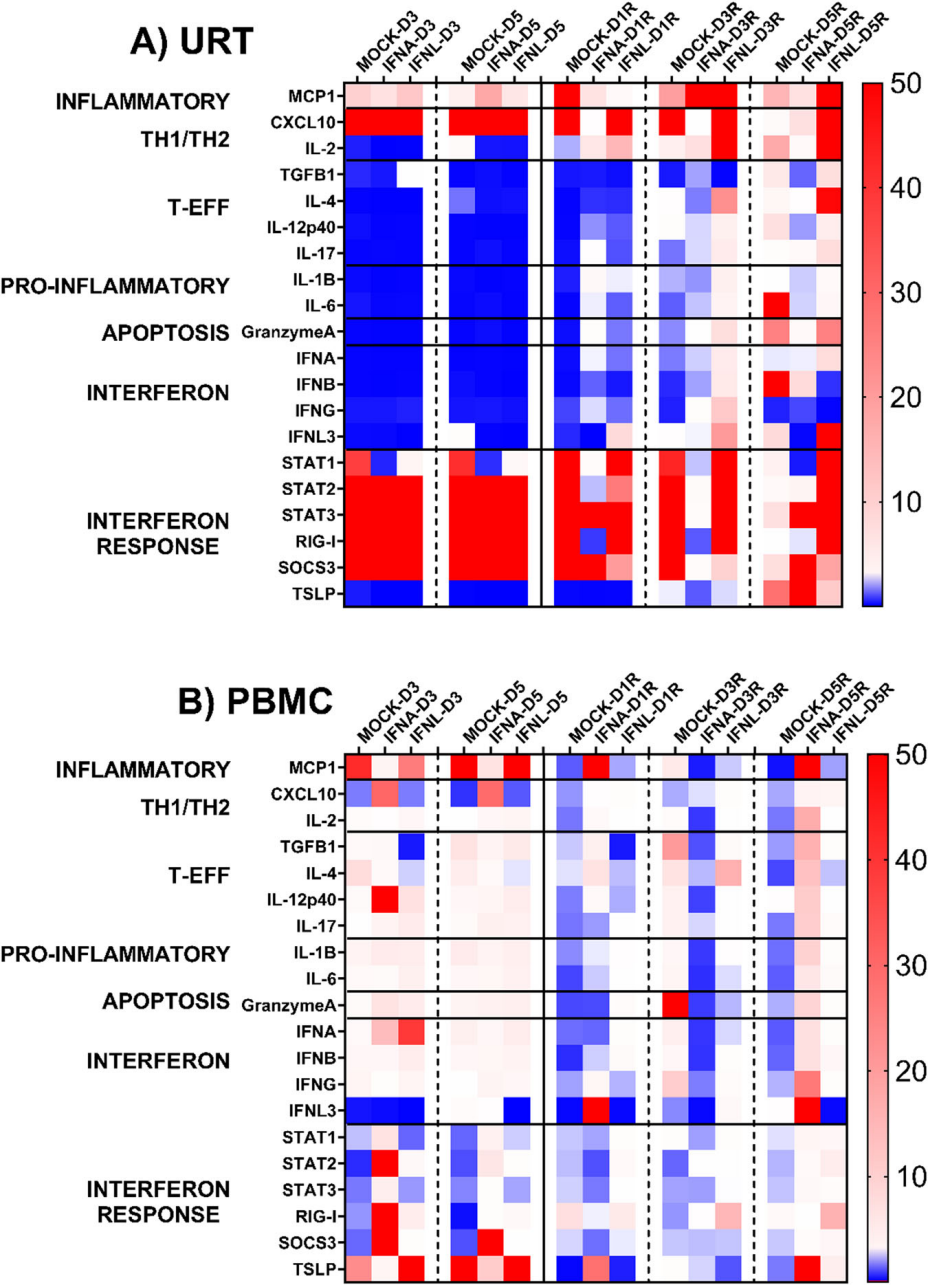
Gene expression in IFN-treated ferret URT and PBMC following IBV challenge-rechallenge. Heatmap of average fold gene expression in IBV-challenged and re-challenged ferrets following IFN-treatment. Animals were challenged with IBV (B/Phuket/3073/2013) and treated with IFNA, IFNL, or MOCK on D1 post Challenge. D3 and D5 post challenge (Dx) are indicated on the left side of each panel and arranged by treatment group. IFNA, IFNL, or mock-treated animals were re-challenged (DxR) with B/Phuket/3073/2013 3 months post challenge. D1, D3, and D5 post re-challenge (DxR) are indicated on the right side of each panel and arranged by treatment group. Genes were normalized to GAPDH (Gene expression = 1). Upregulated genes (>1) are in red and down-regulated genes (<1) are in blue. **A** Average fold gene expression in NW cells of the URT following IBV challenge (Dx) or re-challenge (DxR). **B** Average fold gene expression in PBMC following IBV challenge (Dx) or re-challenge (DxR).

Differential gene expression by qRT-PCR was performed on cells of the URT (NW cells) and PBMC following IBV challenge and treatment with IFNs (Fig. 2). For samples collected from the URT following challenge or rechallenge, all groups showed high levels of TH1 (*CXCL10)* gene expression following challenge; however, only Mock and IFNL treated animals showed upregulation following rechallenge. Interestingly, following rechallenge, Mock treated animals had the highest gene expression of interferon response (*SOCS3*) early on D1 and D3 whereas IFNL treated animals demonstrated later late expression on D5. Only IFNL treated animals showed and upregulation of IFNL3 gene expression following rechallenge (Fig. 2A). No detectable IFN genes (*IFNA*, *IFNB*, *IFNG*, and *IFNL3*) were upregulated in any ferrets in the URT at D3 or D5 post-challenge. Concurrently, *SOCS3* was upregulated in all ferrets. Upon rechallenge at 3 months an early upregulation of TH1 (*CXCL10*), TH2-directed response^44^ (*IL-2*), T-effector (*IL-17*), apoptosis (*Granzyme A*), pro-inflammatory (*IL-1B*), and IFN (*IFNL3*) genes was detected in IFN-treated ferrets and was sustained through D5 post rechallenge. IFNL-treated ferrets had, in general, strong upregulation of all genes examined following rechallenge.

There was upregulation of type-I/II IFNs at D3 and type-I/II/III IFNs at D5 in PBMC collected from IFN-treated ferrets (Fig. 2B). Type-I IFN expression levels at D3 and D5 were lower in mock-treated ferrets. There were equal levels of type-II IFN at D3 and type-III IFN at D5 in IFNL-treated animals. For inflammatory (*MCP-1*) and pro-inflammatory (*IL-1B*) genes, the responses were upregulated early (D3) in IFN-treated ferrets and peaked late (D5) in mock-treated ferrets. Following rechallenge at 3 months post-treatment, inflammatory, TH1, TH2 directed, IFN, and TSLP genes were all upregulated early (D1) in IFN-treated ferrets and sustained through D5. In contrast, upregulation of the same responses in mock-treated ferrets occurred at (D3), which was only transiently expressed and down-regulated by D5. IFN (*IFNL3*) gene was only upregulated at D5 (1.3-fold) in mock-treated ferrets. IFN-treated ferrets had the highest overall gene upregulation post challenge and D3/D5 post rechallenge. This overall expression was sustained following rechallenge in IFN-treated ferrets, whereas in mock-treated ferrets, overall gene expression was only transiently upregulated (D3) following rechallenge.

### Serum cytokines and chemokines levels are elevated in IBV-infected ferrets following IFN treatment

**Fig. 3:**
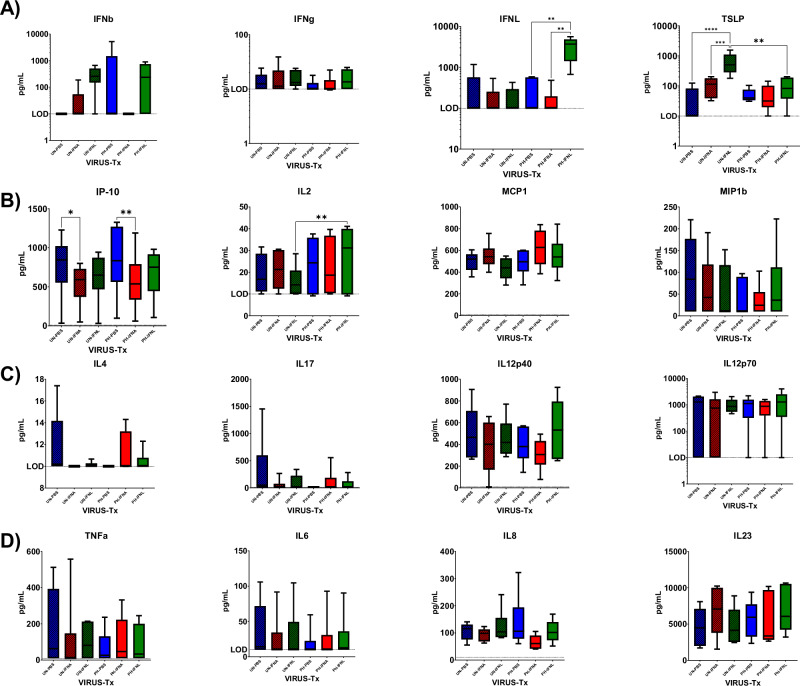
Cytokine and chemokine levels in sera of IFN-treated ferrets following IBV challenge. Serum cytokine and chemokine levels in IBV-infected ferrets following IFN-treatment. Mock-infected groups (UN-X) are represented by hatched colored bars and IBV-challenged groups (PH-X) are indicated by solid colored bars. Treatment groups are Mock (PBS, blue), IFNA (red) and IFNL (green). Average range ±SD of cytokines are presented over 12 weeks post challenge/mock-challenge with average indicated by black line within each bar. Average pG/mL levels of analytes are shown from the following functional groups: **A** Interferon responses. Type-I (IFNB), Type-II (IFNG) and Type-III* (IFNL) and Type-III IFN response cytokine (TSLP). **B** TH1 (IP-10) and TH2 (IL-2) directed responses. **C** Pro-inflammatory chemokines (MCP-1 and MIP-1B). **D** T-effector response (IL-4, IL-17, IL-12p40 & IL-12p70). **E** Pro-inflammatory cytokine response (TNFA, IL-6, IL-8, and IL-23). Significant differences indicated between each group by 2-way ANOVA.

Ferrets challenged with IBV or treated with IFNA or IFNL had elevated serum cytokine and chemokine levels (Fig. 3). Type-III IFN (IFNL) levels were significantly higher in IFNL-treated animals following IBV challenge. Concurrently, TSLP was also significantly higher in IFN-L-treated animals. Type-I and Type-II IFNs were higher following IFN-treatment; however, they were not significantly different from mock-treated (Fig. 3A).

TH1 response (IP-10), TH2 directed response (IL-2) and chemokines (MCP-1 and MIP1B) were also elevated in IFN-treated ferrets compared to untreated animals (Fig. 3B). However, TH1 (IP-10) levels were highest in untreated ferrets compared IFN-treated animals in both unchallenged and challenged animals (Fig. 3B) and rechallenged animals (Supplementary Fig. 4: 7R, dotted blue line). These ferrets had inflammatory responses, such as a fever of +3.2 °F temperature over baseline. T-effector responses were higher for IL-4 following IBV-challenge and IFN-treatment early at 1 week post-challenge (Supplementary Fig. 4C). In contrast, these responses were suppressed in untreated Ferrets although the levels were generally low. Cytokines, IP-10 and IL-12p40, associated with influenza severity^45,46^ were only observed in untreated ferrets challenged at 3 months (Supplementary Fig. 4: 7R, dotted blue line). Pro-inflammatory levels were generally suppressed in collected sera following IBV challenge (Fig. 3D) or rechallenge (Supplementary Fig. 4D) except for IL-8 and IL-23, which were highest following IFNA-treatment. Serum IFNs and other cytokines and chemokines were highest in IFN-treated animals, which were consistent with gene expression observed in IFN-treated ferrets.

### IFN treatment did not affect IBV-LAIV vaccine replication, but reduced morbidity in ferrets following IBV challenge

Ferrets were either vaccinated intranasally with live-attenuated influenza vaccine, B/Phuket/3073/2013-CDC-LV11B (LAIV) or mock vaccinated (UN). One day post vaccination, PEG-IFNA, PEG-IFNL or PBS (mock) was administered intranasally (3 ferrets per group). For mock-vaccinated (UN-Tx) ferrets (Table 5A), there was no lethargy or adverse effects of IFN treatment. Following vaccination (LAIV-Tx), there was no lethargy or fever observed, regardless of treatment. Ferrets administered IFNL (LAIV-IFNL) had little weight loss. IFN treatment did not affect LAIV replication in the URT or the virus kinetics following infection between these two sets of ferrets (*p* = ns, 2-way ANOVA).

**Table 5:** Clinical signs and virus replication in ferrets following IBV vaccination and challenge

	Group-Tx^a^		Inactivity^b^ RII	Days sneezing	Average weight loss^c^	Average Fever^d^	NW viral load^*e*^ (Avg±SD Log_10_ FFU/mL)			
			(D1–7)	(%)	(D1–7)	(Max day)	D1	D3	D5	D7
A)→Vaccination		UN-MOCK	1.00	-	0.8%	-	N/A	N/A	N/A	N/A
		UN-IFNA	1.00	-	-	-	N/A	N/A	N/A	N/A
		UN-IFNL	1.00	-	0.1%	-	N/A	N/A	N/A	N/A
		LAIV-MOCK	1.02	-	-	-	2.88 ± 0.11	3.88 ± 0.96	3.31 ± 0.26	1.30 ± 0.00
		LAIV-IFNA	1.04	-	-	-	2.96 ± 0.11	4.26 ± 0.56	4.06 ± 0.96	1.72 ± 0.73
		LAIV-IFNL	1.00	-	1.2%	-	3.24 ± 0.21	5.17 ± 0.24	3.48 ± 0.37	1.68 ± 0.67
B)→Challenge (3MO)		UN-MOCK	1.25	D2–D4 (33)	2.8%	+4.4 °F (D2)	5.72 ± 0.59	5.55 ± 0.17	4.21 ± 0.66	1.72 ± 0.28
		UN-IFNA	1.01	-	4.1%	+3.5 °F (D2)	5.19 ± 0.99	5.52 ± 0.33	3.83 ± 0.34	1.53 ± 0.39
		UN-IFNL	1.01	D3 (33)	1.4%	+2.3 °F (D2)	5.53 ± 0.76	5.35 ± 0.32	4.31 ± 0.51	2.08 ± 0.26
		LAIV-MOCK	1.00	-	-	-	2.06 ± 0.61	1.47 ± 0.30	2.06 ± 0.67	1.90 ± 0.43
		LAIV-IFNA	1.01	-	-	-	1.54 ± 0.42	1.74 ± 0.38	2.93 ± 0.33	2.36 ± 0.62
		LAIV-IFNL	1.00	-	1.2%	-	2.07 ± 0.88	2.02 ± 0.87	1.60 ± 0.38	1.92 ± 0.53

Clinical signs and virus replication in the URT of ferrets vaccinated with live-attenuated influenza vaccine (B/Phuket/3073/2013-CDC-LV11B; “LAIV”) followed by IFN treatment (or Mock) on D1 post vaccination. Group designated as “UN” were mock vaccinated and groups designated as “LAIV” were vaccinated. A) Initial vaccination clinical signs and virus replication (of LAIV). Groups above the dotted line were mock vaccinated with PBS and groups below the dotted line were vaccinated with LAIV. NW viral loads listed as N/A for mock-vaccinated groups. B) At 3 months post vaccination, all groups were challenged with PH.

^a^Relative inactivity index “RII” (D1–D7) post challenge. RII=${\sum }_{(D1-7)}\left[{score}+1\right]n/{\sum }_{(D1-7)}n$.

^b^Day post challenge of peak weight loss percentage (day) from pre-vaccination or pre-challenge weight from D1–D7 post vaccination or challenge.

^c^Average elevated temperature of at least +2 °F (Fever) above baseline (days post challenge elevated). Fever indicated by any temperature greater than the average baseline (D-2 to D0 pre-challenge) +2 °F.

^d^Average viral load in ferret URT on D1 through D7 post challenge. Baseline = 1.30 Log_10_ FFU/mL.

IFNL treated animals showed modest weight loss following influenza virus challenge at 3 months post-vaccination. No ferrets had clinical signs of infection (Supplementary Fig. 2E, F) and little virus was detected in the NWs post challenge (Supplementary Fig. 2G, H). No differences in virus kinetics were observed between the two sets of ferrets (ns, 2-way ANOVA).

### IFN treatment induced early IFN and inflammatory responses in ferret URT, but not in PBMC following IBV-LAIV vaccination, but IFNL response was sustained following challenge

**Fig. 4:**
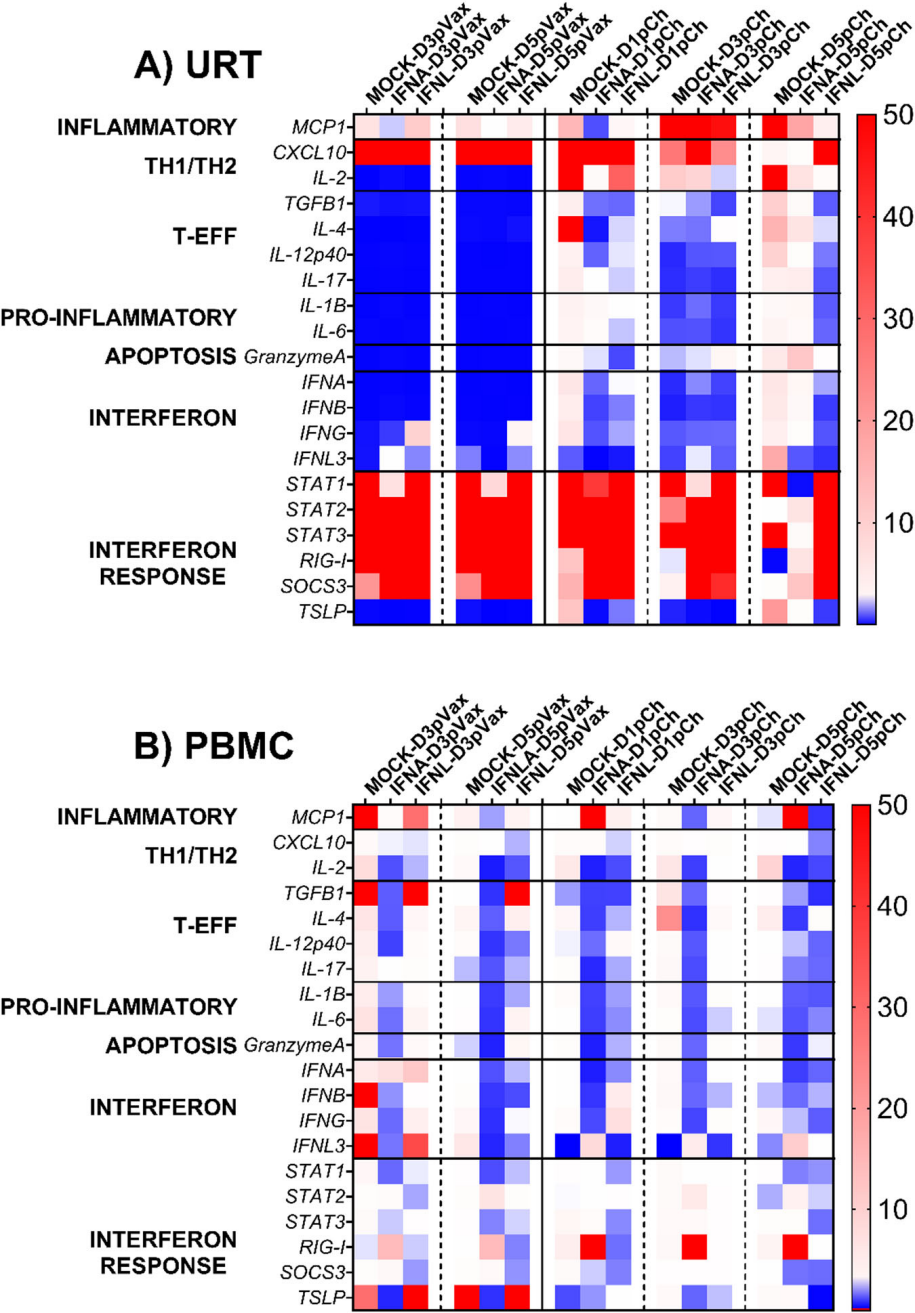
Gene expression in ferret URT and PBMC following IBV vaccination and challenge. Heatmap of average fold gene expression in IBV-vaccinated and challenged ferrets following IFN-treatment. Animals were vaccinated with IBV-LAIV and treated with IFNA, IFNL, or MOCK on D1 post vaccination. D3 and D5 post vaccination (pVax) are indicated on the left side of each panel and arranged by treatment group. IFNA, IFNL or mock treated animals were challenged with B/Phuket/3073/2013 3 months post vaccination. D1, D3, and D5 post challenge (pCh) are indicated on the right side of each panel and arranged by treatment group. Genes were normalized to GAPDH (Gene expression = 1). Upregulated genes (>1) are in red and down-regulated genes (<1) are in blue. **A** Average fold gene expression in NW cells of the URT following IBV vaccination (pVax) or challenge (pCh). **B** Average fold gene expression in PBMC following IBV vaccination (pVax) or challenge (pCh).

Differential gene expression by qRT-PCR was performed on cells of the URT (NW cells) and PBMC following IBV-LAIV vaccination and treatment with IFNs (Fig. 4). IFNA-treated ferrets had the highest expression of TH1 (*CXCL10* gene) and interferon response (*SOCS3* gene) in NW cells from the URT following vaccination and challenge compared to ferrets administered IFNL- or mock ferret groups (Fig. 4A). These results were similar to the gene expression profiles in ferrets infected with B/Phuket/3073/2013 (PH) in the challenge-rechallenge ferret groups. Type-I/II IFN genes were all downregulated, however, type-II(*IFNG*) and type-III (*IFNL3*) were upregulated in IFNL-treated and IFNA-treated ferrets at D3 and D5 post-vaccination. The *SOCS3* gene was upregulated in all ferrets following vaccination and challenge. IFN-treated ferrets showed persistent, highly upregulated TH1 (*CXCL10*) and IFN-response (*SOCS3*) genes following challenge. Pro-inflammatory (*MCP1)* was upregulated by D5 post vaccination and D3 post challenge in IFNA-treated ferrets but sustained in mock-treated ferrets throughout vaccination and challenge.

Unlike the challenge-rechallenge study, there is an upregulation of only type-I IFNs at D3 in the PBMC collected from IFNA-treated ferrets and following vaccination (Fig. 4B). Type-III IFN (*IFNL3*) and IFN response (*TSLP*) genes were upregulated only in IFNL-treated and mock-treated ferrets post vaccination. Other inflammatory, such as TH1/TH2 directed and IFN (*IFNB*, *IFNG*) genes, were expressed at the highest levels in mock-treated ferrets. Following challenge at 3 months post-vaccination, IFN-treated ferrets had high and sustained expression of inflammatory (*MCP1*), type-III IFN (*IFNL3*), IFN response (*RIG-I* and *TSLP*) compared to mock-treated ferrets that had sustained expression of TH1/TH2, T-effector, and IFN (*IFNA*) genes. Unlike the sustained upregulated expression of inflammatory and IFN genes in IFN-treated animals following IBV challenge-rechallenge, responses following LAIV-vaccination and challenge had a weaker inflammatory response in IFN-treated ferrets. However, type-III IFN (*IFNL3*) and inflammatory (*MCP1*) genes were sustained in IFN-treated animals. Interestingly, mock-treated vaccinated ferrets exhibited earlier inflammatory responses post-challenge compared to mock-treated rechallenged ferrets in the challenge-rechallenge study. In the previous study, mock-treated rechallenged ferrets had a transient inflammatory response on D3.

### IFN treatment did not enhance serum cytokine and chemokine levels following IBV-LAIV vaccination and challenge in ferrets

**Fig. 5:**
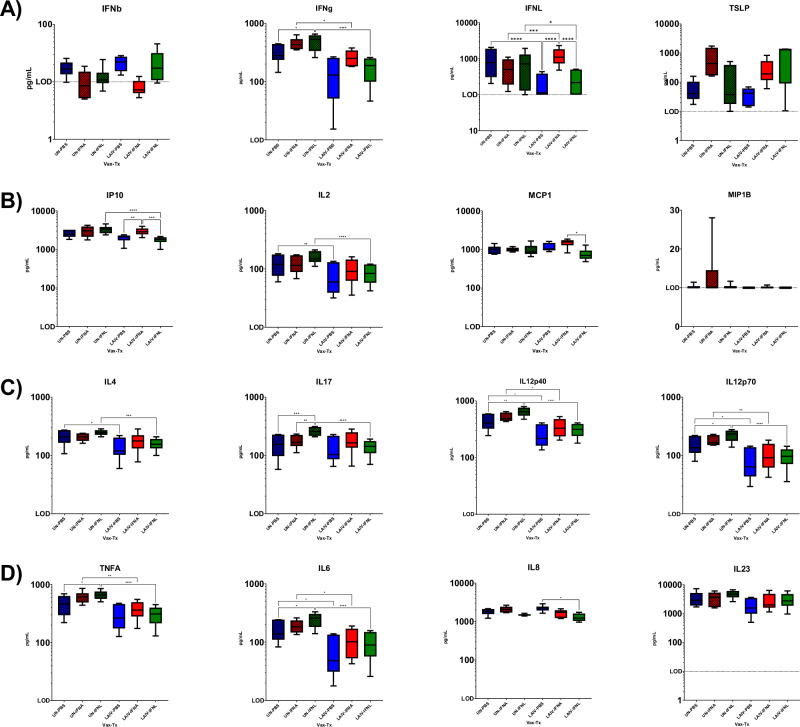
Cytokine/chemokine levels in sera of IFN-treated ferrets following IBV vaccination. Serum cytokine and chemokine levels in IBV-vaccinated ferrets following IFN-treatment. Unvaccinated groups (UN-X) are represented by hatched colored bars and IBV-vaccinated groups (LAIV-X) are indicated by solid colored bars. Treatment groups are Mock (PBS, blue), IFNA (red) and IFNL (green). Average range ±SD of cytokines are presented over 12 weeks post vaccination with average indicated by black line within each bar. Average pG/mL levels of analytes are shown from the following functional groups: **A** Interferon responses. Type-I (IFNB), Type-II (IFNG) and Type-III* (IFNL) and Type-III IFN response cytokine (TSLP). **B** TH1 (IP-10) and TH2 (IL-2) directed responses. **C** Pro-inflammatory chemokines (MCP-1 and MIP-1B) and T-effector response (IL-4, IL-17, IL-12p40, and IL-12p70) (**D**) Pro-inflammatory cytokine response (TNFA, IL-6, IL-8, and IL-23). Significant differences indicated between each group by 2-way ANOVA.

Serum cytokine and chemokine levels were evaluated in ferrets following IBV-LAIV vaccination and treatment with IFNA or IFNL3 for 12Wk and 1Wk following challenge (Fig. 5). Analyte levels were normalized to pre-vaccination (D0) levels to determine whether cytokine and chemokines were either increased or decreased from pre-vaccination levels. IFN levels were similar to levels detected over a 12-week period following vaccination in all ferrets regardless of treatment (Fig. 5A). However, TSLP levels were consistently higher in IFNA- and IFNL-treated animals. TH1 (IP-10) responses were consistently higher in IFNA-treated ferrets following LAIV-vaccination than IFNL- or mock (PBS)-treated animals (Fig. 5B) that was consistent with the TH1 (*CXCL10)* gene expression responses observed in ferret NW cells. The kinetics of T-effector cytokines in IFN-treated ferrets were similar to mock (Fig. 5C). IFNA- and mock-treated animals had increases in IL-4 and IL-17 within the first 1–2 weeks post-treatment and showed similar increases post challenge, whereas IFNL-treated ferrets had lower levels of both IL-4 and IL-17. Pro-inflammatory cytokines were similar for all ferrets (Fig. 5D). Overall, IFN-treatment did not have an enhanced effect on cytokine and chemokine levels indicated following IBV-challenge.

### IFN treatment following IBV challenge generated higher neutralizing antibody titers compared to IBV alone in ferrets following challenge and rechallenge

Ferrets were infected with IBV (PH) followed by intranasal treatment with PEGylated type-I (IFNA), type-III (IFNL) IFN, or Mock (UN) and assessed for serum neutralizing antibodies to IBV by FRA (Table 6). For challenge-rechallenge study, all ferrets reached geometric mean titers (GMT) of >1:160 (bold) by week 4 that fell below 1:160 threshold by 12 weeks (Table 6A). IFNA- and IFNL-treated animals had higher titers than mock-treated ferrets at week 4 (IFNA/IFNL3 = 296, Mock = 235) and week 8 (IFNA = 296, IFNL = 235, Mock = 202) post-challenge. At 1 week post rechallenge, neutralizing titers in IFNA-Tx ferrets were 1.2 times higher (GMT = 1016) and in IFNL3-treated ferrets it was 1.7 times higher (GMT = 1493) than mock-treated animals (GMT = 871). The effects of IFN-treatment on unchallenged animals were only slightly higher than mock-treated animals following challenge 12 weeks later, however, this did not result in neutralizing titers reaching the 1:160 threshold. IFN-treatment clearly showed higher neutralizing antibody levels for 4–8 weeks following IBV challenge compared to mock. IFN-treatment resulted in significantly higher antibody responses following rechallenge over mock-treatment which may indicate a more robust adaptive immune response.

**Table 6:** Neutralizing antibody responses following IBV challenge or vaccination in ferrets treated with IFN

A) Challenge									
		Treatment^a^							
		Mock			IFNA			IFNL	
		UN-PH^b^	PH-PH^c^		UN-PH^b^	PH-PH^c^		UN-PH^b^	PH-PH^c^
Weeks post challenge	Pre	-	5		-	5		-	5
	1	-	34		-	32*		-	19****
	2	-	148		-	148		-	122****
	4	-	**235**		-	296****		-	296****
	8	-	**202**		-	296****		-	235****
	12	-	101		-	148****		-	86****
Challenge (Rechallenge)	1	93	**(871)**		118****	(1016)****		109****	(1493)****
B) Vaccination									
		Treatment^d^							
		Mock			IFNA			IFNL	
		UN-PH^e^	LAIV-PH^f^		UN-PH^e^	LAIV-PH^f^		UN-PH^e^	LAIV-PH^f^
Weeks post vaccination	Pre	-	5		-	5		-	5
	1	-	86		-	80****		-	63****
	2	-	93		-	127****		-	80****
	4	-	118		-	127****		-	93****
	8	-	137		-	218****		-	187****
	12	-	50		-	93****		-	74****
Challenge	1	137	**10240**		218****	**10240**		218****	7525****

A) Serum neutralizing antibodies by FRA (geometric mean titer) in ferrets challenged with B/Phuket/3073/2013 and rechallenged 3 months (12 weeks) later with homologous virus. ^a^Treatment groups include Mock (PBS), PEGylated-IFNA (IFNA), or PEGylated-IFNL3 (IFNL) on day 1 post challenge. ^b^Mock-challenged group (UN-PH), followed by challenge at 3 months. All animals were treated on day 1. ^c^Challenge-rechallenge group (PH-PH), challenged followed by treatment on day 1 post challenge and rechallenged at 3 months post challenge. Rechallenge titers are indicated within parentheses. Neutralizing titers > 1:160 are indicated in bold. B) Serum neutralizing antibodies by FRA (geometric mean titers) in ferrets vaccinated with B/Phuket/3073/2013-LAIV (LAIV) and challenged 3 months (12 weeks) later with B/Phuket/3073/2013. ^d^Treatment groups include Mock (PBS), PEGylated-IFNA (IFNA), or PEGylated-IFNL3 (IFNL) on day 1 post vaccination. ^e^Mock-vaccinated group (UN-PH), followed by challenge at 3 months. All animals were treated on day 1. ^f^Vaccination-challenge group (LAIV-PH), vaccinated and treated on day 1 post vaccination and challenged at 3 months. Neutralizing titers > 1:160 are indicated in bold. Significance compared to Mock (multiple unpaired *T*-tests) indicated to the right of treatment groups.

### IFN treatment following LAIV vaccination generated higher neutralizing antibodies compared to mock treatment but did not result in higher antibody levels following challenge

Ferrets were vaccinated with LAIV-IBV (LAIV) followed by intranasal treatment with PEGylated type-I (IFNA), type-III (IFNL3) IFN, or Mock (UN) and assessed for serum neutralizing antibodies to IBV by FRA (Table 6). For the vaccination study (Table 6B), ferrets treated with IFN had a geometric mean neutralizing titers (GMT) of >1:160 by 8 weeks post vaccination (IFNA-Tx = 218, IFNL-Tx = 187). Interestingly, in the absence of vaccination (UN-PH), IFN-treated animals had neutralizing antibody titers >1:160 at 1-week post-challenge, even though these animals were treated with IFN 12 weeks earlier. IFN-treated ferrets failed to attain antibody titers greater than mock-treated animals following challenge of vaccinated ferrets. IFN-treatment did not result in higher neutralizing antibodies than mock-treatment following LAIV vaccination.

### Serum TSLP directly correlated and IFN inversely correlated to neutralizing Ab responses in ferrets following IBV challenge-rechallenge or LAIV vaccination-challenge

Next, serum cytokine levels were compared to neutralizing antibody responses in ferrets following IBV challenge or IBV vaccination (Table 7). Correlations, that include 3Mo rechallenge or post vaccination challenge are shown in Supplementary Table 2. There was a direct correlation between TSLP and neutralizing antibody response in both mock-treated (*r* = 0.77) and IFNL-treated (*r* = 0.71) ferrets following IBV challenge-rechallenge (Supplementary Table 2A). There was an inverse correlation between IFNG and neutralizing antibody response following IBV challenge and IFNA-treatment (Table 7A). Additionally, T-effector (IL-12p40) levels, inversely correlated with antibody responses following IBV challenge, regardless of treatment. Only mock-treated and IFNL-treated ferrets had an indirect correlation to antibody levels and pro-inflammatory cytokines (TNFA and IL-6) post-challenge and rechallenge. However, IL-8 was indirectly correlated with antibody responses, regardless of treatment (Supplementary Table 2A).

**Table 7:** Correlations between antibody levels and protein levels in IFN-treated ferrets following IBV challenge and vaccination

				MOCK		IFNA-Tx		IFNL-Tx			
	GROUP	ANALYTE	*r*	*P*-value significance	*r*	*P*-value significance	*r*	*P*-value significance			
A) Antibody titer vs analyte (challenge)		IFNB	−0.6547	0.1667	ns	0.3482	0.3333	ns	−0.5071	0.1667	ns
	INTERFERON	IFNG	0.08571	0.4597	ns	**−0.8827**	**0.0167**	*	−0.5429	0.1486	ns
		IFNL	−0.5071	0.1667	ns	−0.6742	0.1667	ns	0.6571	0.0875	ns
		TSLP^a^	0.6	0.175	ns	0.0513	0.5	ns	0.5	0.225	ns
	TH1/TH2	IP-10	0.4286	0.2097	ns	**0.765**	**0.0444**	*	0.3714	0.2486	ns
		IL-2	−0.3714	0.2486	ns	0.08827	0.4444	ns	−0.4857	0.1778	ns
	CHEMOKINE	MCP-1	0.2	0.3569	ns	**−0.765**	**0.0444**	*	0.4286	0.2097	ns
		MIP-1B	0.1352	0.4	ns	−0.1177	0.4056	ns	−0.3381	0.2667	ns
		IL-4	−0.02899	0.4917	ns	−0.5591	0.1333	ns	−0.03036	0.5	ns
	T-EFF	IL-17			−0.4377	0.1833	ns	−0.4395	0.2	ns	
		IL-12p40	−0.6	0.1208	ns	**−0.765**	**0.0444**	*	−0.6	0.1208	ns
		IL-12p70	−0.7714	0.0514	ns	−0.2648	0.3111	ns	−0.6571	0.0875	ns
		TNFA	**−0.8827**	**0.0222**	*	−0.6866	0.0833	ns	−0.6377	0.1	ns
		IL-6	**−0.8452**	**0.0333**	*	−0.6866	0.0833	ns	**−0.8452**	**0.0333**	*
	PRO-INFLAM.	IL-8	**−0.8286**	**0.0292**	*	−0.5591	0.1333	ns	−0.6	0.1208	ns
		IL-23	−0.2571	0.3292	ns	0.2354	0.3278	ns	−0.6	0.1208	ns

				MOCK		IFNA-Tx		IFNL-Tx			
	GROUP	ANALYTE	*r*	*P*-value significance	*r*	P-value significance	*r*	*P*-value significance			
B) Antibody titer vs analyte (vaccination)		IFNB	0.7714	0.0514	ns	−0.4928	0.1667	ns	0.4857	0.1778	ns
	INTERFERON	IFNG	0.3714	0.2486	ns	0.2029	0.3556	ns	0.2571	0.3292	ns
		IFNL	0.3339	0.2667	ns	**−0.8117**	**0.0361**	*	0.3479	0.2472	ns
		TSLP^a^	−0.1	0.475	ns	**0.8721**	**0.05**	*	0.8	0.0667	ns
	TH1/TH2	IP-10	0.7714	0.0514	ns	0.2029	0.3556	ns	0.08571	0.4597	ns
		IL-2	0.4286	0.2097	ns	0.2319	0.3361	ns	0.2571	0.3292	ns
	CHEMOKINE	MCP-1	−0.02857	0.5	ns	0.4058	0.2167	ns	−0.4857	0.1778	ns
		MIP-1B				−0.6642	0.1667	ns			
		IL-4	0.05798	0.4667	ns	0.3189	0.2778	ns	0.3714	0.2486	ns
	T-EFF	IL-17	0.5429	0.1486	ns	0.3189	0.2778	ns	0.3714	0.2486	ns
		IL-12p40	0.5429	0.1486	ns	0.2319	0.3361	ns	0.2571	0.3292	ns
		IL-12p70	0.4857	0.1778	ns	0.2319	0.3361	ns	0.3714	0.2486	ns
		TNFA	0.3714	0.2486	ns	0.3189	0.2778	ns	0.3714	0.2486	ns
	PRO-INFLAM.	IL-6	0.3714	0.2486	ns	0.2319	0.3361	ns	0.3714	0.2486	ns
		IL-8	−0.08571	0.4597	ns	−0.4058	0.2167	ns	−0.4286	0.2097	ns
		IL-23	0.4286	0.2097	ns	0.2029	0.3556	ns	0.2571	0.3292	ns

Spearman non-parametric correlation (One-tailed, 95% confidence interval) was estimated to determine the relationship between neutralizing antibody response and protein levels in serum. ^a^TSLP listed in interferon group due to its direct regulation by IFNL. A) Antibody correlations in challenge study for Mock-, IFNA- and IFNL-treated groups (3 animals/group). Timepoints tested include pre-challenge; weeks 1, 2, 4, 8, and 12 weeks post challenge. A correlation coefficient of *r* < 0 indicates an inverse correlation of antibody titer to protein levels and an *r* > 0 indicates a direct correlation. Strong positive (*r* > 0.7) or negative (*r* < −0.7) correlations are underlined and significant (*p* < 0.05) correlations are in bold. B) Antibody correlations in vaccination study for Mock-, IFNA- and IFNL-treated groups (3 animals/group). Timepoints tested include pre-vaccination; weeks 1, 2, 4, 8, and 12 weeks post vaccination. A correlation coefficient of *r* < 0 indicates an inverse correlation of antibody titer to protein levels and an *r* > 0 indicates a direct correlation. Significant (*p* < 0.05) correlations in bold.

Correlations of cytokines to neutralizing antibodies are shown in Table 7B that include both 3 months rechallenge or 3 months post-vaccination challenge (Supplementary Table 2B). Only TSLP had a strong direct correlation to neutralizing antibody in IFN-treated ferrets either post vaccination or 3 months post-challenge (Table 7 and Supplementary Table 2B). There was a correlation between neutralizing antibody and IFNL and a strong inverse correlation (*r* = −0.8) to the IFNA-treated ferrets both post-vaccination and vaccination-challenge.

## Discussion

When characterizing the innate immune responses of IAV and IBV infection of ferret primary respiratory tract cells, the expression of pro-inflammatory genes and IFNs was delayed and downregulated by IBV strains compared to IAV strains^36^. Subsequent studies of IAV and IBV infections of ferrets confirmed that a delay in initial innate responses resulted in weak antibody responses to IBV^13^. Due to these observations, the goal of this study was to determine whether addition of IFNs, as potential immunoadjuvants, could overcome the delay in the initial innate immune responses to IBV and boost the antibody response following infection or vaccination.

To boost the immune responses to IBV, PEGylated ferret IFNA and IFNL3 were chosen in both challenge-rechallenge and vaccination-challenge studies. Intramuscular treatment of ferrets with seasonal influenza vaccine plus human PEGylated IFNA2b stimulated innate interferon stimulating genes compared to vaccine alone^41^. A previous study, using intra-muscular treatment of ferrets with influenza vaccine plus PEGylated human IFNA2b (Unitron PEG, Schering-Plough), found that 1 μG/Kg was sufficient to stimulate innate interferon stimulating gene responses to IAV compared to vaccine alone^41^. Previously, the innate immune responses in the URT of ferrets were characterized. In this study, ferret IFNs were introduced intranasally in order to assess the effects directly on cells in the URT and determine the impact on virus replication, as well as downstream adaptive immune responses. Since, live-attenuated influenza vaccines were administered intranasally, IFN was also administered intranasally. By administering both virus and vaccine mucosally, coupled with the fact that IFNL is highly expressed on epithelial cells lining the respiratory tract, the immune responses could be assessed at the site of infection^47,48^. Since IFNs are activated following influenza virus infection and affect influenza virus replication in mice and ferrets^49–51^, initially ferrets were treated with IFN at various times in order to determine the minimal effect on virus replication, while still stimulating innate immune responses. Virus replication was reduced if PEG-IFNs were added within 8 h post-challenge. However, if PEG-IFN was added 24 h or later post-challenge, virus replication was not affected. In this study, following IBV infection in ferrets, IFN treatment resulted in reduced morbidity compared to untreated animals. Although animals treated with IFNL had greater weight loss than untreated ferrets, a clear difference in ferret activity post-challenge and vaccination was observed even in IFNA-treated animals. Human studies with IFN-treatment indicate that weight loss of 12–29% has been observed in 90% of HCV patients^52^. A possible mechanism of underlying weight loss may be due to suppression of appetite due to the induction of TNF by IFN. In our study, IFN-treated animals did show higher levels of weight loss than mock-treated animals; however, there were no differences in TNF levels in sera between IFN-treated and mock-treated animals. For animals challenged with IBV 3 months following IFN treatment, only mock and IFNA-treated ferrets developed fever. This was an interesting observation since animals were only treated once with IFNs more than 3 months prior to challenge; however, some residual effect on the inflammatory response by IFNL may be evident. In fact, Type-III IFN has been shown to be anti-inflammatory^53^; however, the long-term effects of a single treatment have not been shown.

In the URT of IBV challenged animals, IFN-treatment induced early inflammatory responses and increased IFNL expression following rechallenge, but PBMC had high and sustained upregulation of type-I/II/III IFNs following IBV challenge and rechallenge in IFN-treated animals. In the URT, *SOCS3* was upregulated in all ferrets and has been shown to down-modulate IFN signaling in a RIG-I-dependent pathway to inhibit antiviral responses and suppress the inflammatory response^54–56^. Concurrently with IFN responses, only IFN-treated animals had elevated levels of type-I/II/III IFNs throughout infection and following rechallenge. This indicates that IFN-treatment can influence both IFN gene expression and serum cytokines levels following IBV infection that are otherwise suppressed and delayed. Viral infection in the respiratory tract IFNL is required for production of virus-specific IgG1, IgA, as well as generation of antiviral CD8+ T cells^21^. The mechanism behind this response affects IFNL triggering the synthesis of TSLP in upper airway M cells, which in turn influences germinal center responses by migrating DCs, thus allowing for enhancement of mucosal immunity and IAV resistance. Following IFNL-treatment of ferrets following IBV challenge or vaccination, there were increased *IFNL3* gene expression and protein levels that correlated to an increase in *TSLP* gene expression and protein levels in PBMC. Interestingly, IFNA-treatment also increased IFNL3 expression and TSLP protein following rechallenge. This induction may be due to an alternate, unknown mechanism of TSLP activation. Innate immune responses were activated following IFN-treatment that resulted in a strong sustained antibody response for 4–8 weeks following challenge. By week 12 post-vaccination, these ferrets had neutralizing antibody activity less than 1:160 and animals were rechallenged with IBV to observe antibody recall responses and enhancement. For untreated ferrets, antibody titers to IBV increased 8.6-fold from 12-week levels (GMT from 1:101 to 1:871), which was 1 week after re-challenge. IFNA-treated animals had higher antibody titers following rechallenge, however, these levels only increased 6.9-fold (GMT from 1:148 to 1:1016) from 12-week levels. IFNL-treated ferrets had the greatest increase in antibody responses (17.4-fold), from a GMT of 1:86 to 1:1493) following rechallenge. Both IFNA and IFNL-treated animals demonstrated significantly higher neutralizing antibody titers compared to untreated animals.

For the vaccine-challenge study in ferrets, as in the challenge-rechallenge study, vaccine replication was not affected by IFN treatment. All ferrets were protected from challenge at 12 weeks post-vaccination, regardless of treatment during the time of vaccination. However, mock-vaccinated animals treated with IFN did show less morbidity than untreated ferrets, even though a single IFN treatment was given 12 weeks prior to challenge. Unfortunately, no differences in virus replication were observed in any of the ferrets. This indicates IFN may be used prophylactically to prevent increased morbidity following influenza infection. Prophylactic treatment of people with type-I or type-III IFN reduced the severity of influenza virus and other respiratory virus-induced disease^57–59^. Following viral challenge, IFN-treated ferrets had high and sustained gene expression of *IFNL3* and *TSLP* in collected PBMC. However, IFN-treatment did not enhance cytokine effects that may be due, in part, to the protective responses elicited by the vaccine. Further exploration of the residual effects of IFN-treatment need to be determined in challenge studies at time points beyond 12 weeks post-vaccination. Since the vaccine efficacy to IBV has been shown to be lower in children than adults, it would be of interest to determine whether different localized immune responses and cytokine profiling in adults and children contribute to these responses. It has been shown in the ferret model to influenza that there are differences in inducible bronchus-associated lymphoid tissues (iBALT) in adult and newly-weaned young ferrets following IAV infection^60^. Both CCL19 (T-cell activation)^61^ and CXCL13 (B-cell recruitment and germinal center formation following influenza infection)^62^ were significantly upregulated in the lungs of young ferrets following IAV-infection and may result in regulation of both T and B cells in these animals. Future studies to determine whether IFN-treatment could enhance iBALT formation and chemokine expression in these sites following IBV infection or vaccination of ferrets would further support immune differences observed in this study.

There was a strong correlation between TSLP levels and antibody responses following IFN-treatment of ferrets in both challenge-rechallenge and vaccination-challenge studies. This indicates a possible biomarker for assessment of antibody responses following influenza infection or vaccination. Since TSLP responses are upregulated in conjunction with IFNL at D3–D5 in PBMC following IBV infection, TSLP responses could be used as a tool in predicting antibody outcome. Further analysis with different influenza vaccines is necessary to determine whether these markers could be used to assess vaccine efficacy.

Even though there was a benefit of IFN treatment of 1 μG/kG on the immune responses in ferrets, additional treatments, as well as testing different concentrations and routes, may result in a greater benefit of IFN and generation of a more robust immune response. The development and availability of additional ferret immune reagents, especially antibodies to ferret IgA and ferret cell surface markers, will allow for a more in-depth analysis of the immune responses in the ferret model and aid in the development of improved vaccines and therapeutics.

The initial innate responses, especially the IFN response, following influenza virus infection or vaccination are important in the development of robust adaptive immune responses to protect from subsequent infection or lessen severity of disease. As with influenza viruses, other viruses are impaired by IFN signaling, and IFN treatment is a powerful therapeutic^63–66^. Since one of the strongest predictors of a robust antibody response following influenza vaccination is the upregulation of IFN signaling genes (reviewed in ref. ^67^), the importance of this study cannot be understated. While we have shown that IFN-treatment does significantly enhance the neutralizing antibody titers to IBV following infection and vaccination, we have not shown whether this effect can protect from cross-lineage infection by increasing the breadth of the antibody response. Further investigations on the effects of interferon treatment on antibody breadth and cross-lineage protection are warranted and could expand the use of IFN as an immunoadjuvant to future influenza vaccines. Due to the anti-inflammatory characteristics of type-III IFNs and lower toxicity in humans^68^, IFNL may be advantageous over IFNA, which is inflammatory and shows greater adverse effects in humans^69,70^. SARS-CoV-2 studies indicated that lower antiviral type-I and type-III expression and elevated IL-6 expression contributed to development of COVID-19 disease^65^, suggesting the use of IFNA/B and IFNL^71^ or JAK/STAT inhibitors^72^ as potential therapeutics. Consequently, IFN-treatment, especially IFNL, may be an important consideration for use following influenza virus infection or as a potential immunoadjuvant to increase immunity to influenza (especially for IBV) and other viral infections.

## Supplementary information

Supplementary Files

## Data Availability

All data files have been made publically available at Open Science Framework entitled Rowe NPJ 2024 as well as provided as supplemental files to this manuscript online at https://www.nature.com/npjvaccines/.
