# Supplementary material for: Interferon as an immunoadjuvant to enhance antibodies following influenza B infection and vaccination in ferrets
Source: NPJ Vaccines. 2024 Oct 24;9:199. doi: 10.1038/s41541-024-00973-2 (PMC11502657; doi:10.1038/s41541-024-00973-2)
Supplement: Supplementary file 1 — Supplementary Files [file 41541_2024_973_MOESM1_ESM.pdf]

Supplementary Table 1: Primers and probes for ferret genes.

GENE TARGET	FORWARD PRIMER 5'→3'	REVERSE PRIMER 3'→5'	PROBE: (5' CHEMISTRY), <MODIFIED BASE>, (3' CHEMISTRY)	USE	REFERENCE	FUNCTION
GAPDH	TGCGGCCAAGGCAGTAG	AGGCCATGCCAGTGAGCTT		SYBR green	Carolan et al.	Housekeeping gene
MCP1	GCAGCAAGTGTCCCAAAGAAG	GACTGGGGTCAGCGCAGAT	FAM-ATCCTCAAGAC<LNA A>TTCCT-BHQ1	TaqMan	Carolan et al.	Inflammatory
CXCL10	CCTGGCTTCACCGAGTTCT	AGTAGCAGCCCATGGAGTAAAA		SYBR green	Maines et al.	Th1
IL-2	GTTAAAAATTATGAGAGCCCCAGGA	TTGAGTTCTTCTGCTAGACATTGAAGA	FAM-CTAC<LNA A>TGCCCAAGAAG-BHQ1	TaqMan	Carolan et al.	Th2
TGFB1	CGTGCGGCAGCTCTATATT	GCAGAAATTGGCGTGGTAAC	FAM-AAGGATCTGGGCTGGAAGTGG<LNA A>ATC-BHQ1	TaqMan	Rowe et al.(2024)	Treg
IL-4	CCAACAGATTGCTCAGAGGACTT	CACCGAACAGGTCATGTTTGC	FAM-CAGGAACCTC<LNA A>GGAACAT-BHQ1	TaqMan	Carolan et al.	Teff
IL-12p40	GGTGCTATTCACAAGCTCAAGTATG	GGTTTGATGATGTCCCTGATGA	FAM- TACACCAGC<LNA A>GCTTC-BHQ1	TaqMan	Carolan et al.	Teff
IL-17	GGACGGTAAACTACCACATGAACTC	AGACTCCCTTCGCAGAACCA	FAM-TCCCC<LNA A>TCCAGCAAGA-BHQ1	TaqMan	Carolan et al.	Teff
IL-1B	CCTGGTGCTGTATAACTCGTATGAG	TTGGTTCACACTAGTTCCGTTGA	FAM-TCGGGCGCTCC<LNA A>C- BHQ1	TaqMan	Carolan et al.	Pro-inflammatory
IL-6	GCAGAGAACAACCTAAATCTTCCAA	TGATTGAATTGAGACTGGAAGCA	FAM-CTGGC<LNA A>GAAGAGGAC-BHQ1	TaqMan	Carolan et al.	Pro-inflammatory
Granzyme A	GGATCCTCCCTCTCCCTAAGAA	CCCAGCCTGCAACTTGACA	FAM-ATG<LNA A>TGTCAAACCCGAAAC-BHQ1	TaqMan	Carolan et al.	Apoptosis
IFNA	TCCATCCTGAGGAACTACTTCCAG	AGGCACAAGGGCTGTATTGC	FAM-GAATCTCCCTCT<LNA A>TCTGC-BHQ1	TaqMan	Carolan et al.	IFN (Type I)
IFNB	ATATTTCTCCACCACGGTTCTTG	ACTCCACACTGCTGCTGCTTAG	FAM-AACTATAACTT<LNA A>CTTCGATTCCA-BHQ1	TaqMan	Carolan et al.	IFN (Type I)
IFNG	AACTGGAGAGAGGAGAGTGACAAAA	GTCTTCCTTGATGGTATCCATGC	FAM-TCTCCTTCT<LNA A>CTTGAAACTGT-BHQ1	TaqMan	Carolan et al.	IFN (Type II)
IFNL3	CCAGCCCTGCCTTAAGTTATT	CCTTCCTGTTTACTTGTGCATATTG	FAM-ATGAAACCAG<LNA A>GTGCTGACCCAAA-BHQ1	TaqMan	Rowe et al. (2024)	IFN (Type III)
STAT1	AGCCTTGCATGCCAACTCA	ACAGTCCAGCTTCACCGTGAA		SYBR green	Fang et al.	IFN response
STAT2	AGCTGCTGAAGGAGCTGAAG	TGCCTTCCTGGAGTCTCACT		SYBR green	Fang et al.	IFN response
STAT3	CAACCCCAAGAACGTGAACT	AGCCCACGTAATCTGACACC		SYBR green	Fang et al.	IFN response
RIG-I	AGAGCACTTGTGGACGCTTT	TGCAATGTCAATGCCTTCAT		SYBR green	Fang et al.	IFN response
SOCS3	GCTGGTGCATCACTACATGC	GACCGTCTTCCGACAGAGAT		SYBR green	Fang et al.	IFN response
TSLP	GTCTGGGCACATAACTCTAAGG	CACCCTGGTGTCTCACTAAAC	<FAM>CAGGCCTTGC<LNA A>GATATAGAGCCGATT<BHQ1>	TaqMan	Rowe et al.(2024)	IFN response

List of qRT-PCR primers. All primers used in this study are referenced. Forward (5' to 3'), reverse (5' to 3') and probes are listed. Probes using TaqMan enzyme include special chemistry at the 5'-end (FAM) 3'-end (BHQ1) and internally modified bases (LNA A) that were specifically designed for this study to enhance binding and specificity for ferret genes.

Supplementary Figure 1: PEGylation of ferret IFNs and biological activity

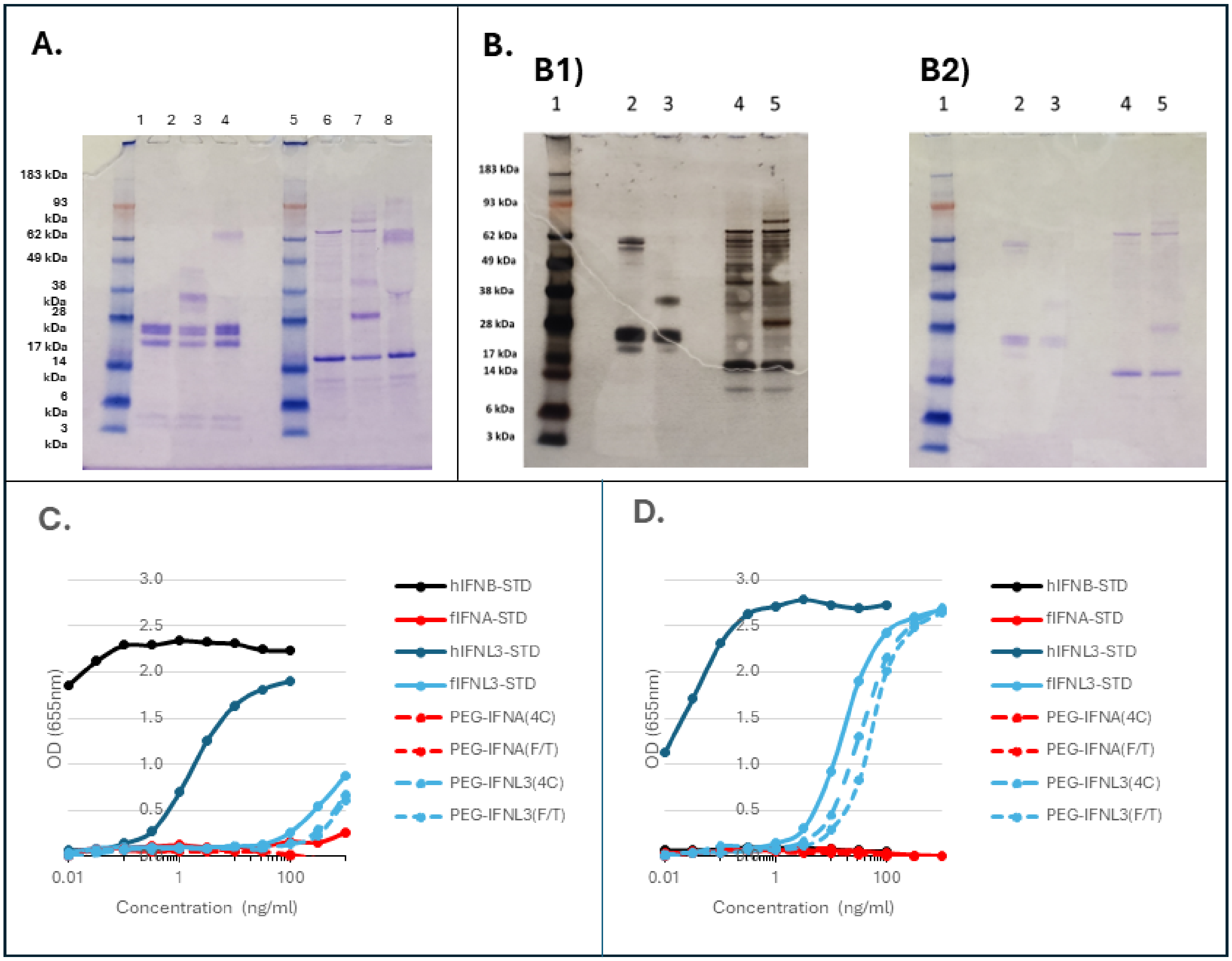

A)Comparison of PEGylation of ferret IFNA and IFNL3 by PEG-5K and PEG-20K; imperial staining. Lane designation: MW standards (1 and 5), ferret IFNA [2 (IFNA), 3 (IFNA+PEG-5K), 4 (IFNA+PEG-20K)], ferret IFNL3 [6 (IFNL3), 7 (IFNL3+PEG-5K), 8 (IFNL3+PEG-20K)] by imperial staining. 2 uG/lane added.

B)Comparison of imperial and silver staining of (PEG-5K) PEGylation of ferret IFNA and IFNL3. Silver staining (B1) and imperial staining (B2). Lane designation: mw standards (1), ferret IFNA [2 (IFNA), 3 (IFNA+PEG-5K)], ferret IFNL3 [6 (IFNL3), 7 (IFNL3+PEG-5K)]. 1 uG/lane added.

C)Type-I IFN bioassay to determine retention of activity of ferret PEGylated IFNA (IFNA+PEG-5K) and stored at 4°C or -80°C (freeze/thaw; “F/T”). Human IFNB standard (solid black line), ferret IFNA standard (solid red line) and PEGylated IFNA stored at 4°C [PEG-IFNA(4C), long red dashed line] or stored at -80°C and thawed [PEG-IFNA(F/T), short red dashed line]. Human IFNL standard (solid dark blue line), ferret IFNL3 standard [fIFNL3-STD, solid light blue line] and PEGylated IFNL3 stored at 4°C [PEG-IFNL3(4C), long light blue dashed line] or stored at -80°C and thawed [PEG-IFNA(F/T), short, dashed light blue line].

D) Type-III IFN bioassay to determine retention of activity of ferret PEGylated IFNL3 (IFNL3+PEG-5K) and stored at 4°C or -80°C (freeze/thaw; “F/T”). Human IFNB standard (solid black line), ferret IFNA standard (solid red line) and PEGylated IFNA stored at 4°C [PEG-IFNA(4C), long red dashed line] or stored at -80°C and thawed [PEG-IFNA(F/T), short red dashed line]. Human IFNL standard (solid dark blue line), ferret IFNL3 standard [fIFNL3-STD, solid light blue line] and PEGylated IFNL3 stored at 4°C [PEG-IFNL3(4C), long light blue dashed line] or stored at -80°C and thawed [PEG-IFNA(F/T), short, dashed light blue line].

Supplementary Figure 2: Weight and temperature kinetics following IBV challenge or vaccination and IFN-treatment in ferrets.

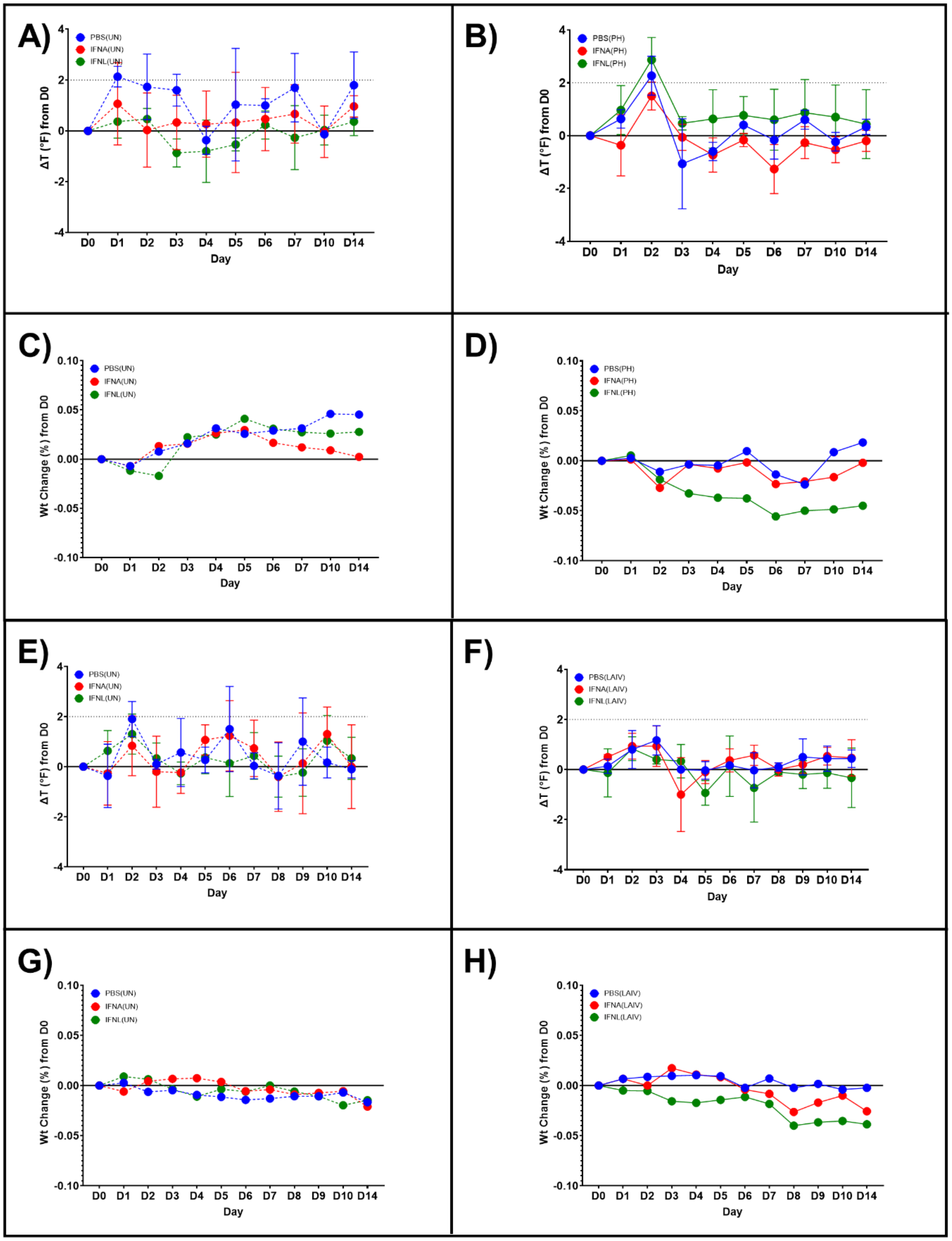

A – D) Challenge experiment temperature and weight changes from D0 to D14 post challenge. A) Average Change in temperature in MOCK-infected ferrets (dotted lines) treated with IFN on day 1 post mock-challenge. Groups are as follows: Untreated (blue). IFNA-treated (red), IFNL-treated (green). Dotted line at +2°F over baseline indicates fever threshold. B) IBV-infected ferrets (solid lines) treated with IFN on day 1 post challenge. Groups are as follows: Untreated (blue). IFNA-treated (red), IFNL-treated (green). Dotted line at +2°F over baseline indicates fever threshold. C) Average weight changes in ferrets mock-challenged (dotted lines) compared to day 0 weights. Changes in weight range from -10% to +10%. Untreated (blue). IFNA-treated (red), IFNL-treated (green). D) Average weight changes in ferrets challenged with IBV (solid lines) compared to pre-challenge weights. Changes in weight range from -10% to +10%. Untreated (blue). IFNA-treated (red), IFNL-treated (green).

E – H) Vaccination experiment temperature and weight changes from D0 to D14 post vaccination. E) Average Change in temperature in Mock-vaccinated ferrets (dotted lines) treated with IFN on day 1 post mock-vaccination. Groups are as follows: Untreated (blue). IFNA-treated (red), IFNL-treated (green). Dotted line at +2°F over baseline indicates fever threshold. F) IBV-vaccinated ferrets (solid lines) treated with IFN on day 1 post vaccination. Groups are as follows: Untreated (blue). IFNA-treated (red), IFNL-treated (green). Dotted line at +2°F over baseline indicates fever threshold. G) Average weight changes in ferrets mock-vaccinated (dotted lines) compared to day 0 weights. Changes in weight range from -10% to +10%. Untreated (blue). IFNA-treated (red), IFNL-treated (green). H) Average weight changes in ferrets vaccinated with IBV (solid lines) compared to pre-vaccination weights. Changes in weight range from -10% to +10%. Untreated (blue). IFNA-treated (red), IFNL-treated (green).

Supplementary Figure 3: Gene expression in ferret URT and PBMC following IFN-treatment.

		[a]URT (NW)								[b]PBMC							
		D3				D5				D3				D5			
GROUP	GENE	MOCK	IFNA	IFNL	[c]EXPRES.	MOCK	IFNA	IFNL	[c]EXPRES.	MOCK	IFNA	IFNL	[c]EXPRES.	MOCK	IFNA	IFNL	[c]EXPRES.
INFLAMMATORY	*MCP1*	2.71	42.11	7.264	IFNA	3.328	1.167	3.198	MOCK	3.092	7.936	446.7	IFNL	14.69	8.359	3.285	MOCK
TH1/TH2	*CXCL10*	0.2228	3.919	1.303	IFNA	0.7754	34.4	1.062	IFNA	1.955	0.7998	0.8035	MOCK	0.7354	0.8882	0.9767	
	IL-2	9.508	6.657	2.202	MOCK	1.685	0.7676	2.769	IFNL	3.707	0.6393	0.7743	MOCK	19.63	0.7783	0.9671	MOCK
T-EFF	*TGFB1*	1.131	1.453	1.556	IFNA~IFNL	0.4563	0.2063	1.834	IFNL	1.593	65.54	69.99	IFNL	53.46	187	124.9	IFNL
	IL-4	5.387	3.113	1.379	MOCK	0.8735	0.7883	2.722	IFNL	2.783	0.7887	1.361	MOCK	29.36	0.462	1.67	MOCK
	IL-12p40	1.06	0.6088	0.8169	MOCK	0.7461	0.308	15.81	IFNL	1.532	0.5599	1.215	MOCK	8.573	18.21	1.317	IFNA
	IL-17	0.6795	0.7769	0.944		1.812	0.4869	0.4969	MOCK	9.48	0.4135	0.3472	MOCK	16.27	16.9	0.808	MOCK~IFNA
PRO-INFLAMMATORY	*IL-1B*	1.333	0.8983	1.235	MOCK~IFNL	4.688	0.4597	0.5722	MOCK	5.093	0.6117	0.6093	MOCK	10.73	1.365	1.348	MOCK
	IL-6	0.5549	1.239	1.211	IFNA~IFNL	0.7236	0.3945	0.8582		2.398	0.3832	0.6974	MOCK	8.29	0.2778	0.9716	MOCK
APOPTOSIS	*GranzymeA*	0.8606	2.31	1.623	IFNA	1.195	0.6679	0.491	MOCK	10.7	0.3259	0.3165	MOCK	9.014	1.934	1.064	MOCK
INTERFERON	*IFNA*	0.8752	1.238	1.026	IFNA	1.137	0.5508	0.503	MOCK	4.812	0.5724	0.739	MOCK	12.25	0.4962	1.113	MOCK
	IFNB	0.8238	1.076	0.6866	IFNA	1.183	0.3363	0.7095	MOCK	2.835	0.2784	0.6375	MOCK	10.27	12.63	0.9961	IFNA
	IFNG	0.6957	1.832	0.9243	IFNA	0.2261	0.4121	1.249	IFNL	1.397	0.8925	1.524	IFNL	13	0.9891	1.165	MOCK
	IFNL3	1.954	3.324	2.513	IFNA	3.525	1	236.8	IFNL	1.021	1.018	1322	IFNL	10.23	1.477	170.8	IFNL
INTERFERON RESPONSE	*STAT1*	0.0977	17.93	17.02	IFNA	2.132	906.5	3200	IFNL	2.014	0.7505	0.5504	MOCK	1.128	0.9228	0.7195	MOCK
	STAT2	0.9613	3.518	0.9951	IFNA	0.4093	88.04	2.604	IFNA	1.401	0.9501	0.6264	MOCK	0.3628	0.7776	1.425	IFNL
	STAT3	1.2	4.516	1.111	IFNA	2.869	1525	3.243	IFNA	1.54	0.6657	0.5695	MOCK	1.507	1.041	0.7678	MOCK
	RIG-I	1.238	3.113	1.397	IFNA	0.3738	21.46	1.916	IFNA	1.321	1.136	0.4259	MOCK	0.7054	0.9187	1.068	IFNL
	SOCS3	0.2403	2.643	2.007	IFNA	5.453	57.9	0.3345	IFNA	4.332	1.152	0.3378	MOCK	1.534	1.215	0.8168	MOCK
	TSLP	0.7344	0.6283	3.533	IFNL	0.053	0.1274	139.3	IFNL	0.6892	1.772	767.9	IFNL	3.993	0.02407	545.7	IFNL

Average fold gene expression in ferrets following IFN-treatment. [a]NW cells from the URT of mock-challenge/vaccinated (PBS) animals and treated with IFNA, IFNL or mock on D1 post challenge. [b]PBMC from animals mock-challenge/vaccinated (PBS) and treated with IFNA, IFNL or mock on D1 post challenge. [c]Expression (EXPRES.) indicates treatment group with highest gene expression increase at each given timepoint post challenge. Gene upregulation (positive fold-increase in gene expression over D0) indicated in pink and down-regulation of gene expression indicated in blue. Target gene functional groups are listed under GROUP and separated by dotted lines.

Supplementary Figure 4: Cytokine/chemokine levels in sera of IFN-treated ferrets following IBV vaccination and challenge.

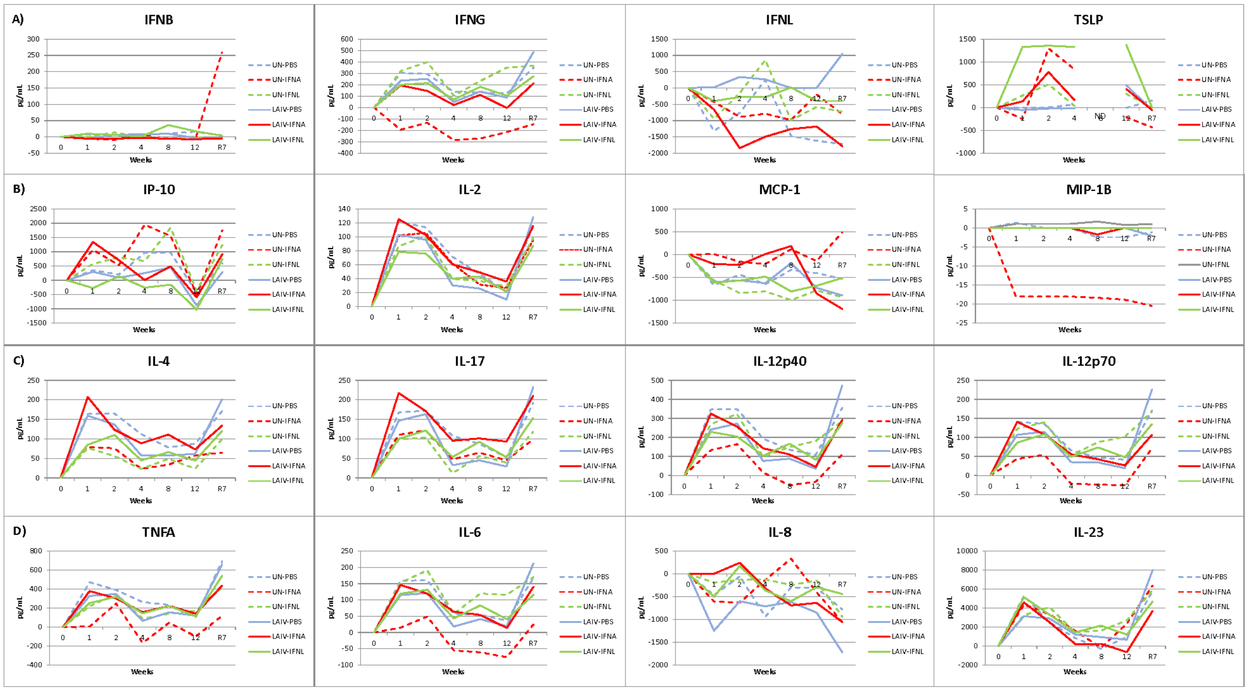

Serum cytokine and chemokine levels in IBV-vaccinated ferrets following IFN-treatment. Mock-vaccinated groups (UN-X) are represented by dotted lines and IBV-vaccinated groups (LAIV-X) are indicated by solid lines. Treatment groups are Mock (PBS, light blue), IFNA (red) and IFNL (green). The x-axis indicated the weeks post vaccination (1, 2, 4, 8, 12) and after 3MO [day 7 (R7) post challenge]. Average pG/mL levels of analytes are shown from the following functional groups: A) Interferon responses. Type-I (IFNB), Type-II (IFNG) and Type-III* (IFNL) and Type-III IFN response cytokine (TSLP). B) TH1 (IP-10) and TH2 (IL-2) directed responses. C)Pro-inflammatory chemokines (MCP-1 and MIP-1B) D)T-effector response (IL-4, IL-17, IL-12p40 & IL-12p70) E)Pro-inflammatory cytokine response (TNFA, IL-6, IL-8, and IL-23). All levels of cytokines/chemokines are normalized to D0 and represent average increases or decreases from pre-vaccination over a 12Wk period post-vaccination plus 1Wk (R7) post-challenge.

Supplementary Table 2: Correlation between antibody levels and protein levels in IFN-treated ferrets following IBV challenge and vaccination – Including 3-month challenge/rechallenge timepoint.

A) CHALLENGE - RECHALLENGE

A) ANTIBODY TITER VS ANALYTE (CHALLENGE-RECHALLENGE)

		MOCK			IFNA-Tx			IFNL-Tx		
**GROUP**	**ANALYTE**	**r**	**P-VALUE**	**SIGNIFICANCE**	**r**	**P-VALUE**	**SIGNIFICANCE**	**r**	**P-VALUE**	**SIGNIFICANCE**
**INTERFERON**	IFNB	-0.6124	0.1429	ns	0.1134	0.4762	ns	-0.5345	0.119	ns
	IFNG	-0.3214	0.2488	ns	-0.5092	0.1246	ns	-0.6847	0.0508	ns
	IFNL	-0.5345	0.119	ns	-0.6236	0.1429	ns	0.6429	0.0694	ns
	TSLP[a]	0.7714	0.0514	ns	0.4638	0.1972	ns	0.7143	0.0681	ns
**TH1/TH2**	IP-10	0.1071	0.4198	ns	0.6183	0.0738	ns	0.07143	0.4532	ns
	IL-2	-0.3929	0.1978	ns	-0.3273	0.2389	ns	-0.5406	0.1067	ns
**CHEMOKINE**	MCP-1	-0.25	0.2974	ns	-0.09092	0.4262	ns	-0.1071	0.4198	ns
	MIP-1B	-0.04454	0.5	ns	-0.4312	0.1643	ns	-0.4009	0.2143	ns
**T-EFF**	IL-4	0.07412	0.4476	ns	-0.6001	0.0825	ns	0.0197	0.5	ns
	IL-17				-0.09436	0.431	ns	-0.49	0.1429	ns
	IL-12p40	**-0.75**	**0.0331**	*	**-0.7456**	**0.031**	*	**-0.75**	**0.0331**	*
	IL-12p70	**-0.8469**	**0.0127**	*	0.03637	0.4778	ns	-0.6429	0.0694	ns
**PRO-INFLAM.**	TNFA	**-0.8982**	**0.0071**	**	-0.4954	0.1325	ns	-0.7042	0.0524	ns
	IL-6	**-0.8018**	**0.0238**	*	-0.4954	0.1325	ns	**-0.8018**	**0.0238**	*
	IL-8	**-0.8929**	**0.0062**	**	**-0.7274**	**0.0365**	*	**-0.75**	**0.0331**	*
	IL-23	-0.3214	0.2488	ns	0.2182	0.3222	ns	-0.5	0.1333	ns

B) VACCINATION - CHALLENGE

B) ANTIBODY TITER VS ANALYTE (VACCINATION-CHALLENGE)

		MOCK			IFNA-Tx			IFNL-Tx		
**GROUP**	**ANALYTE**	**r**	**P-VALUE**	**SIGNIFICANCE**	**r**	**P-VALUE**	**SIGNIFICANCE**	**r**	**P-VALUE**	**SIGNIFICANCE**
**INTERFERON**	IFNB	0.25	0.2974	ns	-0.2342	0.3099	ns	0.2143	0.3308	ns
	IFNG	0.6071	0.0833	ns	0.5045	0.129	ns	0.5357	0.1179	ns
	IFNL	0.593	0.0893	ns	**-0.7928**	**0.0198**	*	0	0.5155	ns
	TSLP[a]	0.3714	0.2486	ns	**0.8407**	**0.0222**	*	**0.8857**	**0.0167**	*
**TH1/TH2**	IP-10	0.6786	0.0548	ns	0.3243	0.2409	ns	0.4286	0.1768	ns
	IL-2	0.6429	0.0694	ns	0.3424	0.2294	ns	0.5357	0.1179	ns
**CHEMOKINE**	MCP-1	-0.3929	0.1978	ns	-0.1261	0.3988	ns	-0.2143	0.3308	ns
	MIP-1B	-0.6124	0.1429	ns	-0.4119	0.2857	ns			
**T-EFF**	IL-4	0.4144	0.1786	ns	0.3964	0.1917	ns	0.6071	0.0833	ns
	IL-17	**0.7143**	**0.044**	*	0.3964	0.1917	ns	0.6071	0.0833	ns
	IL-12p40	**0.7143**	**0.044**	*	0.3424	0.2294	ns	0.5357	0.1179	ns
	IL-12p70	0.6786	0.0548	ns	0.2523	0.2909	ns	0.6071	0.0833	ns
**PRO-INFLAM.**	TNFA	0.6071	0.0833	ns	0.5766	0.0937	ns	0.6071	0.0833	ns
	IL-6	0.6071	0.0833	ns	0.3424	0.2294	ns	0.3214	0.2488	ns
	IL-8	-0.4286	0.1768	ns	-0.6307	0.0706	ns	-0.4286	0.1768	ns
	IL-23	0.6429	0.0694	ns	0.3243	0.2409	ns	0.3571	0.2222	ns

Spearman non-parametric correlation (One-tailed, 95% confidence interval) were estimated to determine the relationship between neutralizing antibody response and protein levels in serum. [a]TSLP listed in interferon group due to its direct regulation by IFNL. A) Antibody correlations in Challenge-Rechallenge study for Mock-, IFNA- and IFNL-treated groups (3 animals/group). Timepoints tested include pre-challenge; weeks 1, 2, 4, 8, 12 weeks post challenge; 1 week post re-challenge. A correlation coefficient of $r<0$ indicates an inverse correlation of antibody titer to protein levels and an $r>0$ indicates a direct correlation. Strong positive ($r>0.7$) or negative ($r<-0.7$)

correlations are underlined and significant ($p<0.05$) correlations are in bold. B) Antibody correlations in Vaccination – Challenge study for Mock-, IFNA- and IFNL-treated groups (3 animals/group). Timepoints tested include pre-vaccination; weeks 1, 2, 4, 8, 12 weeks post vaccination; 1 week post challenge. A correlation coefficient of $r<0$ indicates an inverse correlation of antibody titer to protein levels and an $r>0$ indicates a direct correlation. Significant ($p<0.05$) correlations in bold.
